# Supplementary material for: The Structure of a Conserved Domain of TamB Reveals a Hydrophobic β Taco Fold
Source: Structure. 2017 Dec 5;25(12):1898–1906.e5. doi: 10.1016/j.str.2017.10.002 (PMC5719984; doi:10.1016/j.str.2017.10.002)
Supplement: Document S1. Figures S1–S4 and Table S1 [file mmc1.pdf]

Structure, Volume *25*

Supplemental Information

The Structure of a Conserved Domain of TamB Reveals a Hydrophobic β Taco Fold

Inokentijs Josts, Christopher James Stubenrauch, Grishma Vadlamani, Khedidja Mosbahi, Daniel Walker, Trevor Lithgow, and Rhys Grinter

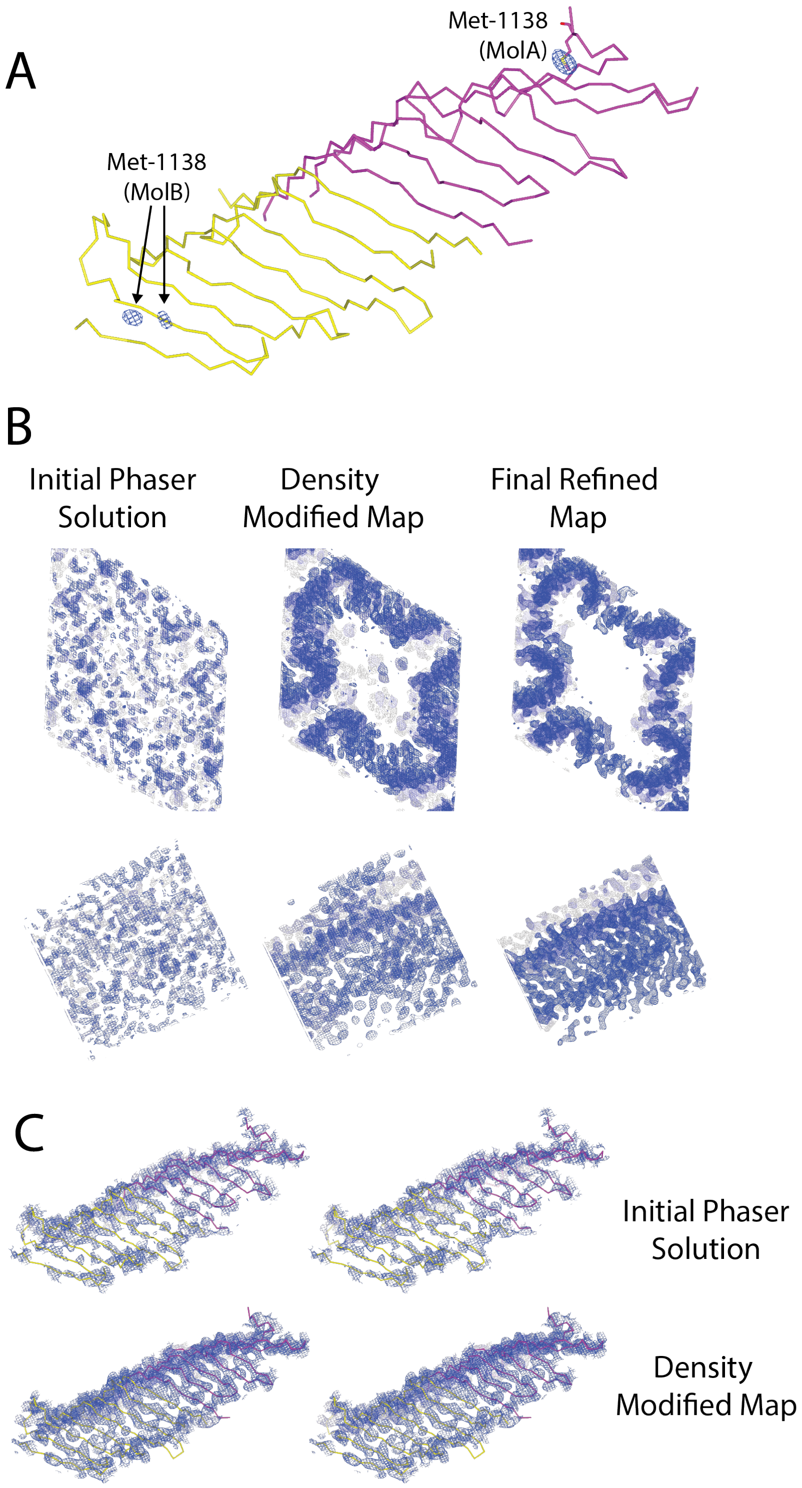

Figure S1, related to Figure 2: Electron density maps from TamB$_{963-1138}$ experimental phasing. (A) positions of selenium sites using for SAD phasing, the two sites attributable to methionine 1138 from molecule B are a result of partial occupancy of this residue. (B) and (C) TamB$_{963-1138}$ electron density maps pre and post density modification, model building and refinement.

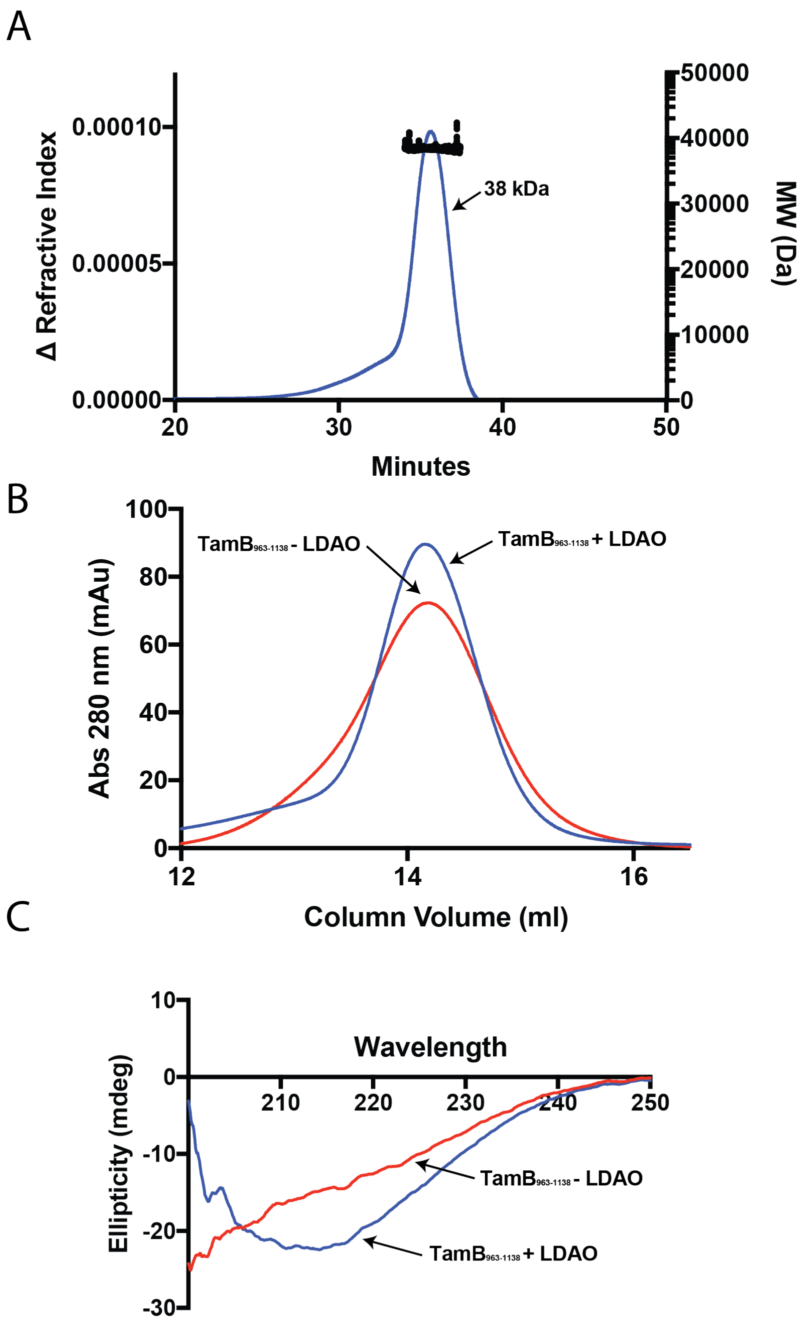

Figure S2, related Figures 2 and 3: Characterisation of TamB$_{963-1138}$ in solution. (A) SEC-MALS analysis of purified TamB$_{963-1138}$ showing that the protein exists as 38 kDa species corresponding to a dimer. (B) Analytical SEC of TamB$_{963-1138}$ in the presence and absence of 0.03 % LDAO. Both proteins elute at a volume corresponding to a dimer. (C) Circular Dichroism analysis of TamB$_{963-1138}$ In the presence and absence of LDAO. In the presence of LDAO TamB$_{963-1138}$ has a minimal ~218 nm, indicative of predominantly β-structure. In the absence of LDAO TamB$_{963-1138}$ has no minima in the far UV range (200-250 nm) suggesting partial disorder.

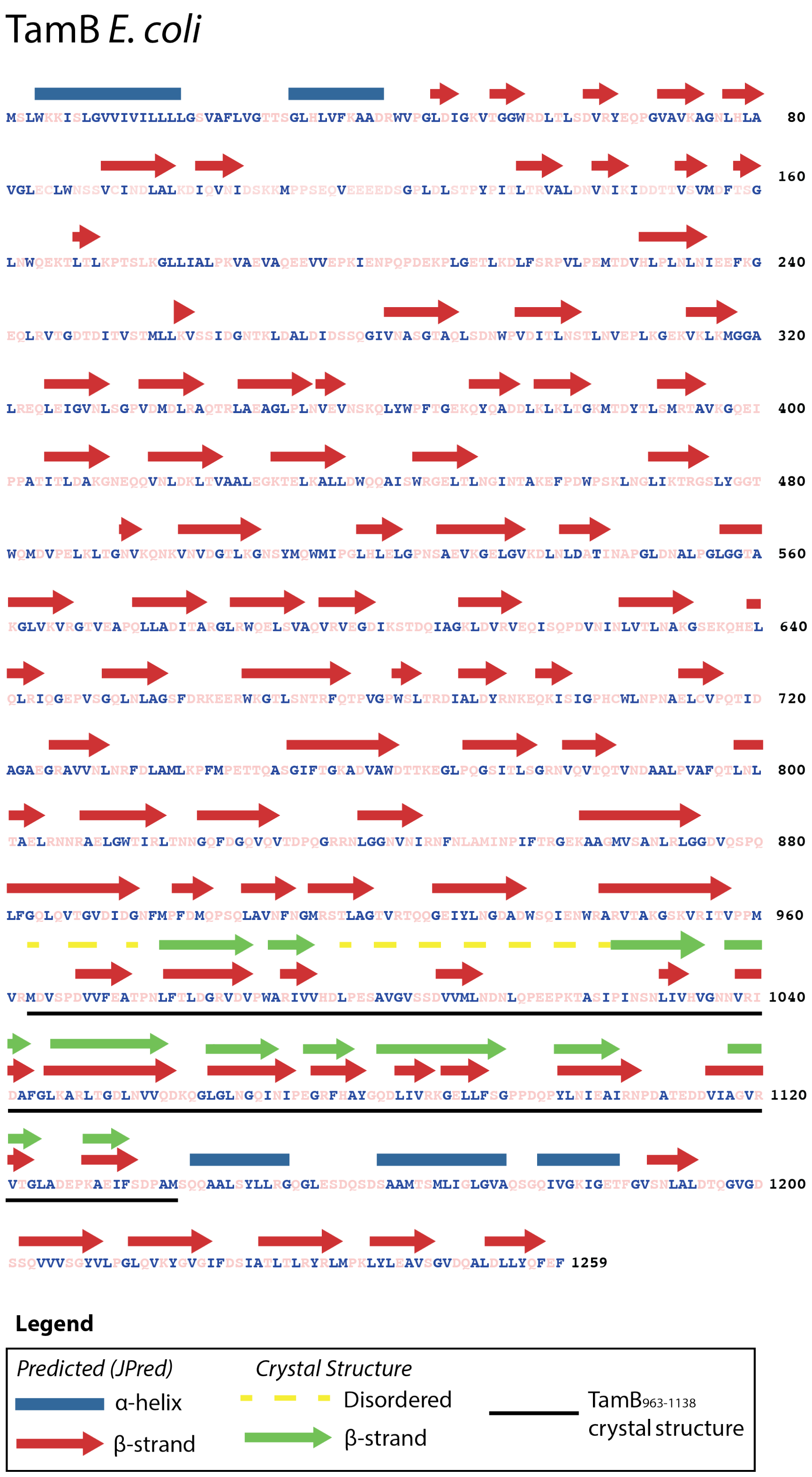

Figure S3, related to Figure 1: Predicted secondary structure of TamB from *E. coli*. TamB secondary structure; Predicted using JPred β-sheet = red arrow, α-helix = blue rectangle. From $TamB_{963-1138}$ crystal structure β-sheet = green arrow, disordered peptide = yellow broken line. The extent of the crystal structure is shown with black underline

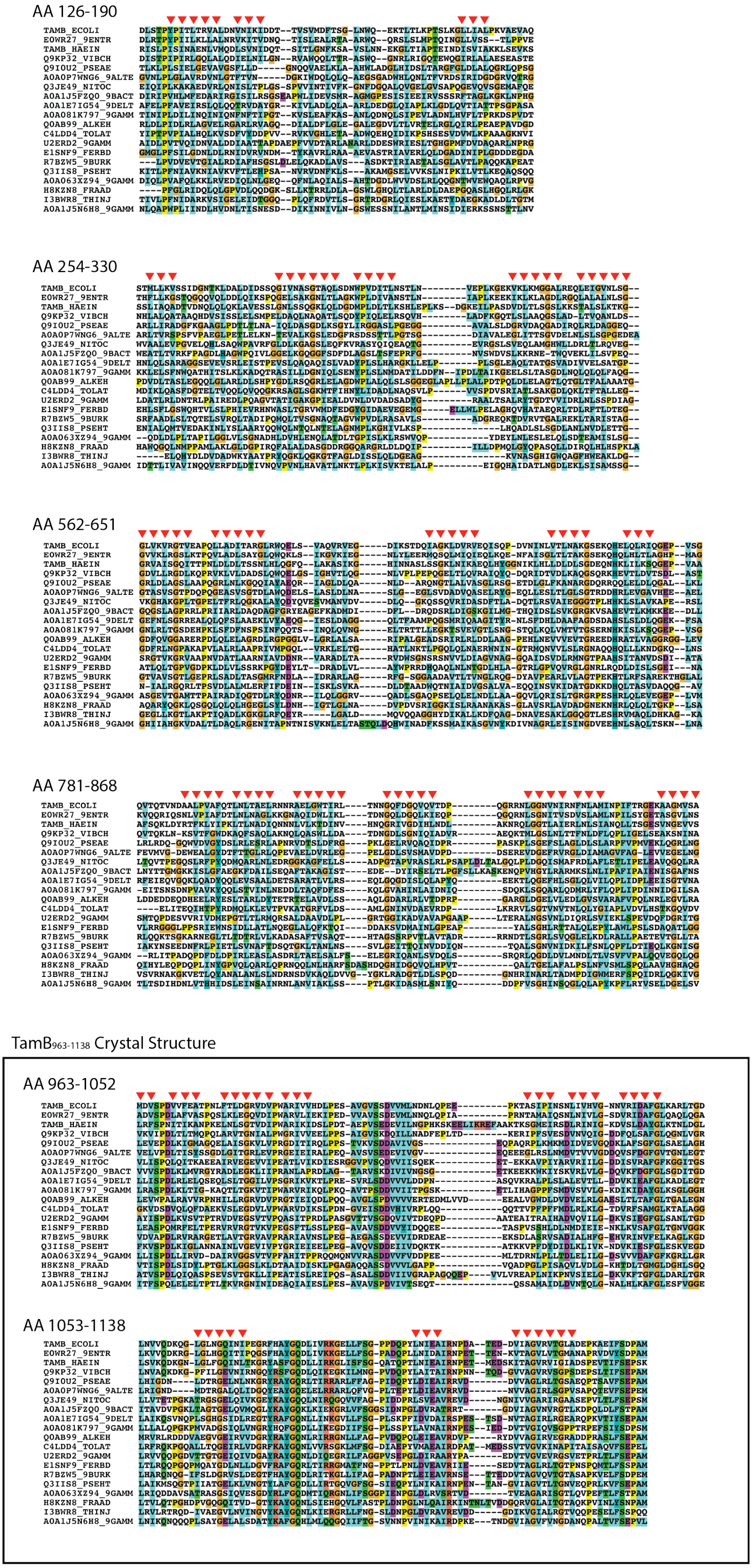
AA 126-190
AA 254-330
AA 562-651
AA 781-868
TamB963-1138 Crystal Structure
AA 963-1052
AA 1053-1138

Figure S4, related to Figure 3: Sequence alignment of sections of TamB homologues showing conserved pattern of alternating hydrophobic residues. See table S1 for details of sequences used for the alignment.

**Identifier**	**Organism**	**Length (AA)**	**E-Value**
TAMB_ECOLI	*Escherichia coli (strain K12)*	1259	1.10E-303
E0WR27_9ENTR	*Candidatus Regiella insecticola LSR1*	1253	3.70E-186
Q9KP32_VIBCH	Vibrio cholerae serotype O1 (strain ATCC 39315 / El Tor Inaba N16961)	1254	3.30E-132
R7BZW5_9BURK	*Sutterella sp. CAG:397*	1273	7.00E-119
TAMB_HAEIN	*Haemophilus influenzae (strain ATCC 51907 / DSM 11121 / KW20 / Rd)*	1298	6.70E-118
A0A081K797_9GAMM	*Endozoicomonas elysicola*	1298	2.50E-97
A0A1E7IG54_9DELT	*Desulfuromonadales bacterium C00003093*	1241	1.40E-94
Q3IIS8_PSEHT	*Pseudoalteromonas haloplanktis (strain TAC 125)*	1225	8.20E-92
Q0AB99_ALKEH	*Alkalilimnicola ehrlichii (strain ATCC BAA-1101 / DSM 17681 / MLHE-1)*	1283	7.20E-83
C4LDD4_TOLAT	*Tolumonas auensis (strain DSM 9187 / TA4)*	1240	1.40E-74
A0A1J5N6H8_9GAMM	*Gammaproteobacteria bacterium MedPE*	1278	3.60E-73
Q9I0U2_PSEAE	Pseudomonas aeruginosa (strain ATCC 15692	1221	8.70E-73
E1SNF9_FERBD	*Ferrimonas balearica (strain DSM 9799 / CCM 4581 / PAT)*	1264	1.60E-72
A0A0P7WNG6_9ALTE	*Marinobacter sp. HL-58*	1246	2.10E-64
Q3JE49_NITOC	*Nitrosococcus oceani (strain ATCC 19707)*	1262	1.20E-52
I3BWR8_THINJ	*Thiothrix nivea (strain ATCC 35100)*	1198	6.10E-52
U2ERD2_9GAMM	*Salinisphaera shabanensis E1L3A*	1297	2.10E-50
A0A1J5FZQ0_9BACT	*Nitrospirae bacterium CG2_30_53_67*	1249	2.30E-46
A0A063XZ94_9GAMM	*Nitrincola lacisaponensis*	1202	6.80E-45
H8KZN8_FRAAD	*Frateuria aurantia (strain ATCC 33424)*	1269	6.70E-42

Table S1, related to Figure 3: Details of TamB homologues used for sequence alignment. E-Value = similarity to sequence of TamB from *E. coli*.
