# Supplementary material for: The Structure of a Conserved Domain of TamB Reveals a Hydrophobic β Taco Fold
Source: Structure. 2017 Dec 5;25(12):1898–1906.e5. doi: 10.1016/j.str.2017.10.002 (PMC5719984; doi:10.1016/j.str.2017.10.002)
Supplement: Document S2. Article plus Supplemental Information [file mmc2.pdf]

Short Article

Structure

The Structure of a Conserved Domain of TamB Reveals a Hydrophobic β Taco Fold

Graphical Abstract

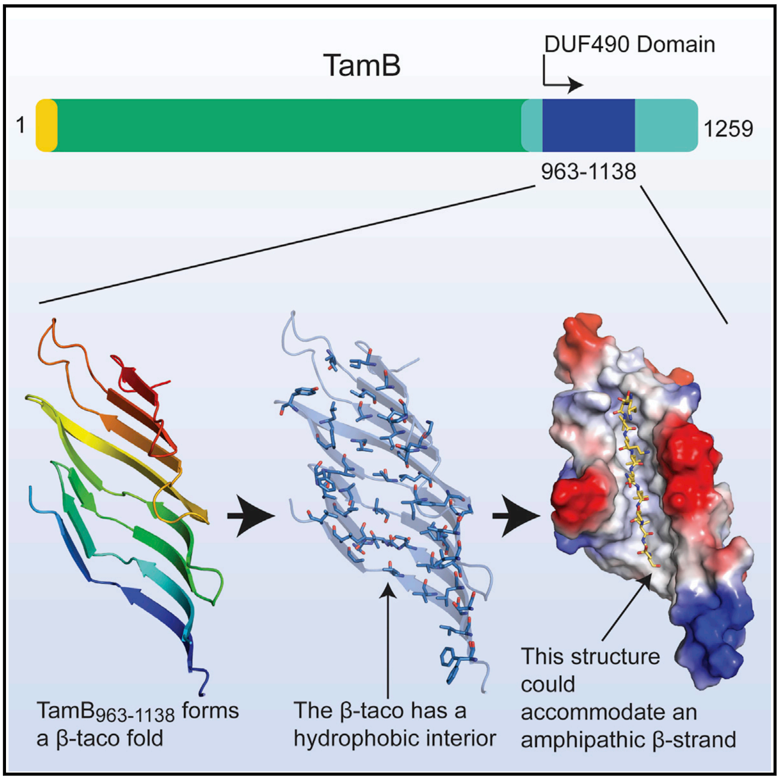

Highlights

- The structure of $TamB_{963-1138}$ reveals a β taco fold with a hydrophobic interior
- The hydrophobic interior of $TamB_{963-1138}$ could chaperone hydrophobic β strands
- Structural analysis suggests the majority of TamB forms a hydrophobic groove

Authors

Inokentijs Josts, Christopher James Stubenrauch, Grishma Vadlamani, Khedidja Mosbahi, Daniel Walker, Trevor Lithgow, Rhys Grinter

Correspondence

rhys.grinter@monash.edu

In Brief

In this work Josts et al. provide structural insight into the bacterial β barrel assembly protein, TamB. This structure suggests that TamB performs its function via a deep hydrophobic groove, capable of accommodating hydrophobic β strands.

Josts et al., 2017, Structure 25, 1898–1906
December 5, 2017 © 2017 The Author(s). Published by Elsevier Ltd.
https://doi.org/10.1016/j.str.2017.10.002

Structure
Short Article

The Structure of a Conserved Domain of TamB Reveals a Hydrophobic β Taco Fold

Inokentijs Josts,[1,2] Christopher James Stubenrauch,[4] Grishma Vadlamani,[4] Khedidja Mosbahi,[3] Daniel Walker,[3] Trevor Lithgow,[4] and Rhys Grinter[4,5,6,*]
[1]The Hamburg Centre for Ultrafast Imaging (CUI), Institute for Biochemistry and Molecular Biology, University of Hamburg, Martin-Luther-King-Platz 6, 20146 Hamburg, Germany
[2]Department of Chemistry, Institute for Biochemistry and Molecular Biology, University of Hamburg, Martin-Luther-King-Platz 6, 20146 Hamburg, Germany
[3]Institute of Infection, Immunity and Inflammation, College of Medical, Veterinary and Life Sciences, University of Glasgow, Glasgow G12 8QQ, UK
[4]Infection and Immunity Program, Biomedicine Discovery Institute and Department of Microbiology, Monash University, Melbourne, VIC 3804, Australia
[5]Institute of Microbiology and Infection, School of Immunity and Infection, University of Birmingham, Birmingham B15 2TT, UK
[6]Lead Contact
*Correspondence: rhys.grinter@monash.edu
https://doi.org/10.1016/j.str.2017.10.002

SUMMARY

The translocation and assembly module (TAM) plays a role in the transport and insertion of proteins into the bacterial outer membrane. TamB, a component of this system spans the periplasmic space to engage with its partner protein TamA. Despite efforts to characterize the TAM, the structure and mechanism of action of TamB remained enigmatic. Here we present the crystal structure of TamB amino acids 963–1,138. This region represents half of the conserved DUF490 domain, the defining feature of TamB. $TamB_{963-1138}$ consists of a concave, taco-shaped β sheet with a hydrophobic interior. This β taco structure is of dimensions capable of accommodating and shielding the hydrophobic side of an amphipathic β strand, potentially allowing TamB to chaperone nascent membrane proteins from the aqueous environment. In addition, sequence analysis suggests that the structure of $TamB_{963-1138}$ is shared by a large portion of TamB. This architecture could allow TamB to act as a conduit for membrane proteins.

INTRODUCTION

In Gram-negative bacteria, the outer membrane (OM) serves as a highly selective permeability barrier, protecting bacterial cells from a hostile external environment, while allowing import of the nutrients required for survival and growth (Silhavy et al., 2010). In addition, the OM forms the interface between the bacteria and its external environment. As such, it plays a pivotal role in the adherence of bacteria to surfaces, as well as in attack and defense (Heinz et al., 2016; Pizarro-Cerdá and Cossart, 2006). To perform this diverse set of functions, the OM contains a multitude of integral membrane proteins (Rollauer et al., 2015). The transport of these proteins from their site of synthesis in the cytoplasm, and their correct and efficient insertion into the OM, poses a significant challenge. Gram-negative bacteria possess a specialized nano-machine termed the β barrel assembly machinery (BAM complex) charged with this task (Noinaj et al., 2013; Webb et al., 2012). In addition, these bacteria possesses the translocation and assembly module (the TAM), a nano-machine which is important in the proper assembly of a subset of OM proteins (Heinz et al., 2015; Selkrig et al., 2012; Stubenrauch et al., 2016a). In the Gram-negative bacterium *Escherichia coli*, the BAM complex contains five (BamA-E) components centered around BamA, an integral OM protein of the Omp85 family (Bakelar et al., 2016; Gu et al., 2016; Han et al., 2016). The TAM is composed of two subunits, TamA an Omp85 family protein evolutionarily related to BamA and the enigmatic inner membrane-anchored protein TamB (Heinz et al., 2015). In *E. coli* and many other Gram-negative bacteria, the presence of BamA is essential for the growth and survival of the cell (Voulhoux et al., 2003; Wu et al., 2005). The TAM on the other hand is dispensable for growth of *E. coli* under lab conditions; however, in a mouse model of infection, TAM mutants from various pathogens exhibit attenuated virulence (Selkrig et al., 2012).

In *E. coli,* TamA and TamB have been shown to associate and, as TamB is embedded in the inner membrane via a signal anchor, it must span the periplasm to interact with TamA (Selkrig et al., 2012). In keeping with this, analysis of recombinant TamB by atomic force microscopy and dynamic light scattering shows it to be highly prolate, with a length of 150–200 Å (Shen et al., 2014). Interaction between TamA and TamB occurs via the conserved C-terminal DUF490 domain of TamB and POTRA1 of TamA and is required for the proper functioning of the TAM *in vitro* (Selkrig et al., 2015; Shen et al., 2014). *In vivo*, the presence of both TamA and TamB is required for the correct assembly of a number of OM proteins (Heinz et al., 2016; Selkrig et al., 2012; Stubenrauch et al., 2016a). In keeping with the role of the TAM in infection, these proteins are predominantly virulence factors, with prominent roles in bacterial adhesion and biofilm formation (Heinz

 © 2017 The Author(s). Published by Elsevier Ltd.
This is an open access article under the CC BY license (http://creativecommons.org/licenses/by/4.0/).

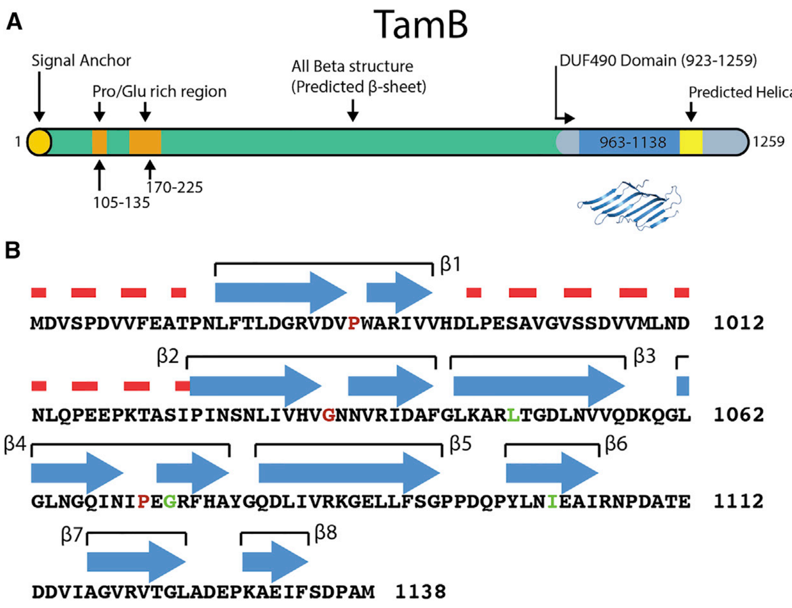

Figure 1. Schematic and Secondary Structure of TamB$_{963-1138}$

(A) Schematic of TamB showing domains, structural elements, and secondary structure.

(B) Sequence and secondary structure of TamB$_{963-1138}$, secondary structure from crystal structure is shown: blue arrows represent β sheets; broken red lines represent residues not resolved in the crystal structure. Residues discussed in text are colored red, and those subjected to mutagenesis are colored green.

et al., 2015; Selkrig et al., 2012; Stubenrauch et al.,2016a). Intriguingly, recent reports have shown that TamB homologs exist even in bacteria that lack TamA (Stubenrauch et al., 2016b; Yu et al., 2017). In *Borrelia burgdorferi*, the causative agent of Lyme disease, TamB has been shown to interact with BamA and appears to be essential for viability (Iqbal et al., 2016). While further investigation is required, these data point toward a more general role for TamB homologs in OM protein biogenesis.

TamB is a large protein by bacterial standards, consisting in *E. coli* of 1,259 amino acids, which are predicted to be composed of predominantly β strand structure (Figure 1A) (Heinz et al., 2015; Shen et al., 2014). To date, no high-resolution structural information on TamB is available and, as no homologs have been structurally characterized, very little information about its structure can be inferred. In this work, we report the crystal structure of TamB$_{963-1138}$ from *E. coli*, a region spanning half of the conserved DUF490 domain (Figure 1B). This structure reveals that TamB$_{963-1138}$ forms a previously undescribed fold, consisting of a concave β sheet with a highly hydrophobic interior, which we refer to as a β taco. We show that TamB$_{963-1138}$ is stabilized by detergent molecules, which likely reside in the hydrophobic cavity of the β taco. Furthermore, sequence analysis of TamB suggests that this structure is shared by the majority of the molecule. Given the role of TamB in the transport and assembly of integral membrane proteins we postulate this hydrophobic cavity may serve as a chaperone and conduit for the hydrophobic β strands of target proteins. This proposed mechanism of TamB has striking similarities to the lipopolysaccharide (LPS) transport system Lpt in which a membrane spanning β jelly roll with a hydrophobic groove is predicted to act as a conduit for LPS (Bollati et al., 2015).

RESULTS AND DISCUSSION

The Crystal Structure of TamB$_{963-1138}$

To gain insight into the structure of the DUF490 domain of TamB, we attempted to crystalize the full-length domain, as well as a number of truncation constructs. One of these constructs, consisting of residues 963–1,138 of TamB (designated TamB$_{963-1138}$) produced diffraction quality crystals and data was collected and anisotropically processed to 1.86–2.2 Å (Josts et al., 2014; Strong et al., 2006). As no homologs of TamB have been structurally characterized, selenomethionine-labeled protein was prepared, crystalized, and the structure was solved using single-wavelength anomalous dispersion (SAD) (Table 1). Substructure solution proved difficult because only weak anomalous signal was present in the data. Despite this, a heavy atom substructure was determined consisting of one high-occupancy site, as well as two low-occupancy sites in close proximity (Figure S1A). Initial SAD phases lacked contrast, making hand determination impossible. However, density modification greatly improved contrast, allowing main-chain tracing (Figures S1B and S1C). This initial model was then used to phase the higher-resolution native data by molecular replacement, and the structure was built and refined (Table S1). The crystal structure of TamB$_{963-1138}$ revealed an elongated taco-shaped molecule consisting entirely of β sheet and random coil. This β taco structure is formed by two molecules of TamB$_{963-1138}$, which interact via their N-terminal β strand to form a continuous 16-stranded curved β structure (Figure 2A). The two molecules of TamB$_{963-1138}$ in this structure consist of eight β strands related by non-crystallographic symmetry. The first of these strands runs parallel to the second, with the subsequent strands adopting an anti-parallel structure (Figure 2B). Between the first and second β strands 29 residues lack electron density due to disorder. This disordered section leads to ambiguity regarding which molecule the first TamB$_{963-1138}$ β strand originates from. Either this first β strand is connected by the disordered loop to the parallel strand of one monomer creating a continuous eight-stranded sheet (Figure 2C), or this loop connects β strand 1 to β strand 2 of the opposing molecule, leading to a β zipper intercalation of the two molecules (Figure 2D). Analysis of purified TamB$_{963-1138}$ in solution by size-exclusion chromatography coupled to multi-angle laser light scatter (SEC-MALS) gave a molecular mass of 38 kDa for TamB$_{963-1138}$. This is twice the 19 kDa mass of an individual TamB$_{963-1138}$ molecule, showing that the crystallography dimer is also the solution state of TamB$_{963-1138}$ (Figure S2A).

Proline residues 987 and 1,071 at the center of β strands 1 and 4 and glycine 1,035 at the center of β strand 2 create a discontinuity which kinks of the β sheet, facilitating the curvature of the

Table 1. $TamB_{963-1138}$ Crystallographic Data Collection and Refinement Statistics

	DUF490 (963–1,138) SelMet	DUF490 (963–1,138) Native
Data Collection[a]		
Space group	$P3_221$	$P3_221$
Cell dimensions		
a, b, c (Å)	57.2,57.2, 220.91	57.24, 57.24, 220.71
α, β, γ (°)	90, 90, 120	90, 90, 120
Wavelength	0.9763	0.9763
Resolution (Å)	73.58–2.69 (2.82–2.69)	49.57–1.86 (1.89–1.86)
R_{merge}	4.2 (64.5)	8.3 (288.7)
R_{pim}	1.1 (19.3)	4.1 (174.0)
$I/\sigma(I)$	46.8 (3.2)	11.3 (0.6)
Completeness (%)	99.0 (94.8)	94.5 (99.2)
Redundancy	17.8 (12.8)	9.2 (6.8)
No. of reflections		35,019 (2,208)
Refinement Statistics		
Anisotropy correction[b]		
Resolution truncation		
a*, b*, c* (Å)		2.2, 2.2, 1.86
Reflections discarded		
Original, discarded, final		34,941, 10,753, 24,188
R_{work}/R_{free}		20.8/25.1
No. of atoms		
Protein		2,091
Waters		131
Ligand/ions		0
RMSD		
Bond lengths (Å)		0.011
Bond angles (°)		1.317

Data from one crystal were collected for each structure. RMSD, root-mean-square deviation.
[a]Values in parentheses are for highest-resolution shell.
[b]Correction applied using the "Diffraction Anisotropy Server" (Strong et al., 2006).

β taco (Figure 2E). The two molecules of $TamB_{963-1138}$ are structurally analogous with a Cα root-mean-square deviation of 0.71 Å. The differences between the molecules is accounted for by flexible loops connecting the β strands; specifically, a large difference in conformation in the loop connecting β strands 6 and 7 (Figure 2F). As $TamB_{963-1138}$ only represents a fragment of the larger TamB, the head-to-head dimer observed in the crystals structure is unlikely to be physiological. However, the oligomeric state of TamB *in vivo* has yet to been definitively determined, so the relevance of this dimer in unknown. The region of TamB N-terminal to $TamB_{963-1138}$ is predicted to consist of a β structure, and so the interaction between the N-terminal strands of the two monomers may act as a surrogate for the β strands of full-length TamB (Figure S3).

The Interior of the $TamB_{963-1138}$ β taco Is Highly Hydrophobic

The most striking feature of the $TamB_{963-1138}$ crystal structure is that the interior surface of its β taco is populated entirely by aliphatic and aromatic residues, making this interior cavity highly hydrophobic (Figures 3A and 3B). During purification of $TamB_{963-1138}$ it was found that the detergent lauryldimethylamine N-oxide (LDAO) was required for stabilization of the domain. Purification of $TamB_{963-1138}$ in the absence of LDAO led the protein to precipitate and resulted in a poor yield of purified protein. $TamB_{963-1138}$ could be purified in the presence of LDAO and, once purified, the protein could be maintained in the absence of the detergent. However, while analytical SEC suggests that $TamB_{963-1138}$ still exists as a dimer under these conditions (Figure S2B), circular dichroism revealed it to be unstructured under these conditions, lacking the characteristic minima for β structured proteins (Figure S2C). Electron density possibly attributable to the aliphatic chains of stabilizing LDAO molecules is evident inside the $TamB_{963-1138}$ cavity (Figure 3C). This density however, is insufficiently resolved to permit accurate modeling of the LDAO head groups and as a result it was not possible to unambiguously attribute it to the detergent. As such, LDAO was not included in the final model submitted to the PDB.

Given the periplasm-spanning topology of TamB, as well as the amphipathic characteristics in the substrate proteins assembled by the TAM, the hydrophobic β taco of $TamB_{963-1138}$ structure is suggestive of a role for TamB in chaperoning membrane proteins across the periplasm to TamA in the OM. The open hydrophobic cleft of $TamB_{963-1138}$ could shield the hydrophobic face of the β strand of an integral membrane protein, while leaving the hydrophilic face exposed to the aqueous environment. In support of this hypothesis, the interior of the $TamB_{963-1138}$ β taco is of a width and depth sufficient to accommodate a single extended β strand (Figure 3D).

To test this hypothesis, we introduced the charged amino acids glutamate or arginine into full-length TamB in the place of Leu_{1049} and Ile_{1102}, respectively. Both these amino acids reside in the $TamB_{963-1138}$ hydrophobic β taco (Figures 4A and 4B). We then tested the ability of these mutant versions of TamB to complement a *ΔtamB E. coli* strain, by observing its function in an established pulse-chase assay, where TAM function is the rate-limiting step in the assembly the fimbrial usher protein FimD (Stubenrauch et al., 2016a). In this assay, proteinase K shaving of the bacterial cell surface is used to detect properly folded, radiolabeled FimD assembled in the OM. Exogenously added proteinase K cleaves FimD (90 kDa) at an extracellular loop, generating a C-terminal (40 kDa) and N-terminal (50 kDa) fragment. However, in the absence of the TAM, a 45 kDa "B fragment" is generated representing a central portion of FimD in a non-native conformation (Stubenrauch et al., 2016a).

Interestingly, placement of an Arg at position 1,102 (Ile_{1102}Arg) significantly impaired the assembly of FimD, leading to the accumulation of the 45 kDa B fragment, indicating that the Ile_{1102}Arg mutant can only partly complement a *tamB* null-phenotype (Figure 4C). Other mutations in the groove had less impact: the ability of the Leu_{1049}Glu mutant to assemble FimD was indistinguishable from wild-type, BN-PAGE analysis of crude membrane

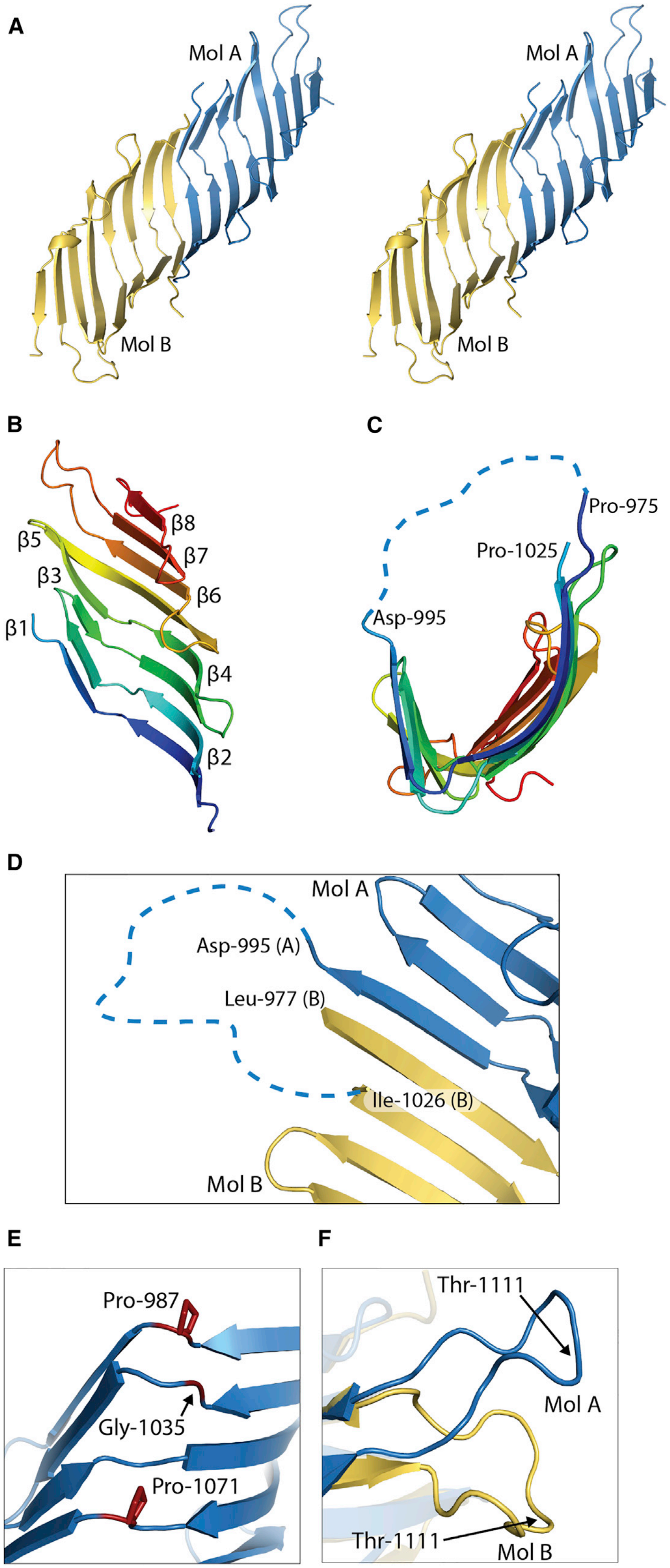

Figure 2. The Crystal Structure of TamB$_{963\text{-}1138}$

(A) Cross-eye stereo view of the TamB$_{963\text{-}1138}$ dimer; molecule A is colored blue and molecule B is colored yellow.

(B) Jones's Rainbow of TamB$_{963\text{-}1138}$, colored from blue (N terminus) to red (C terminus).

(C) TamB$_{963\text{-}1138}$ showing disordered region connectivity option one between Asp$_{995}$ and Pro$_{1025}$ of molecule A.

(D) TamB$_{963\text{-}1138}$ showing connectivity option two between Asp$_{995}$ of molecule A and Ile$_{1026}$ of molecule B.

(E) The kink at the base of the TamB$_{963\text{-}1138}$ β taco is created by Pro$_{987}$ and Pro$_{1071}$ and Gly$_{1035}$.

(F) A large conformational difference is observed in the loop between β strands 6 and 7 of TamB$_{963\text{-}1138}$ molecule A and B.

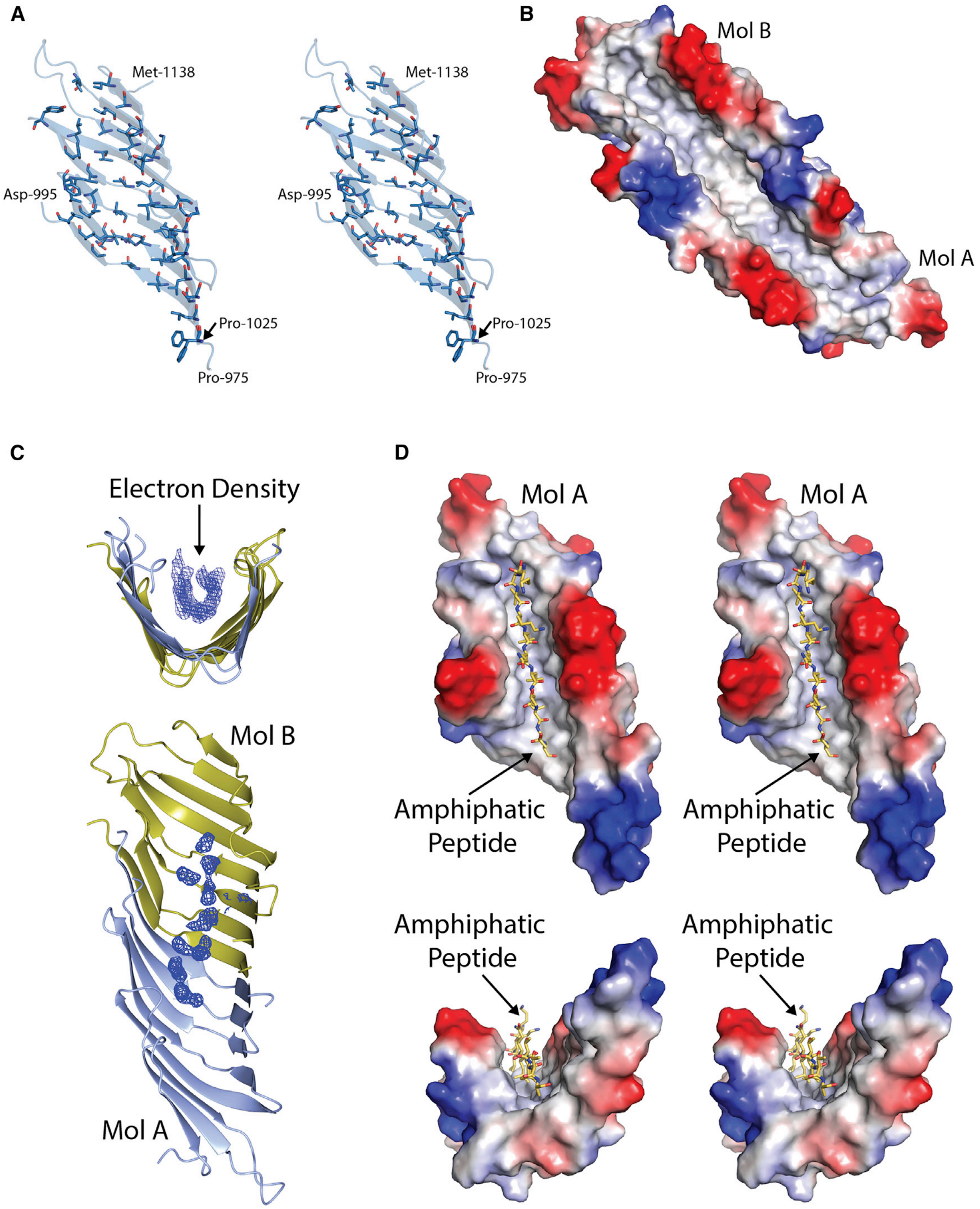

Figure 3. The Interior of the $TamB_{963-1138}$ β Taco Is Hydrophobic

(A) Cross-eye stereo view of $TamB_{963-1138}$ showing as sticks the sidechains facing the interior of the β taco, all sidechains are hydrophobic.

(B) Electrostatic surface model of $TamB_{963-1138}$ molecules A and B, showing the hydrophobic groove.

(C) Electron density present in the $TamB_{963-1138}$ hydrophobic groove attributable to LDAO present in the crystallization buffer. The map presented in a feature-enhanced map generated using the Phenix package, contoured to 1.5 σ (Afonine et al., 2015).

(D) An amphipathic β strand docked into the $TamB_{963-1138}$ hydrophobic groove.

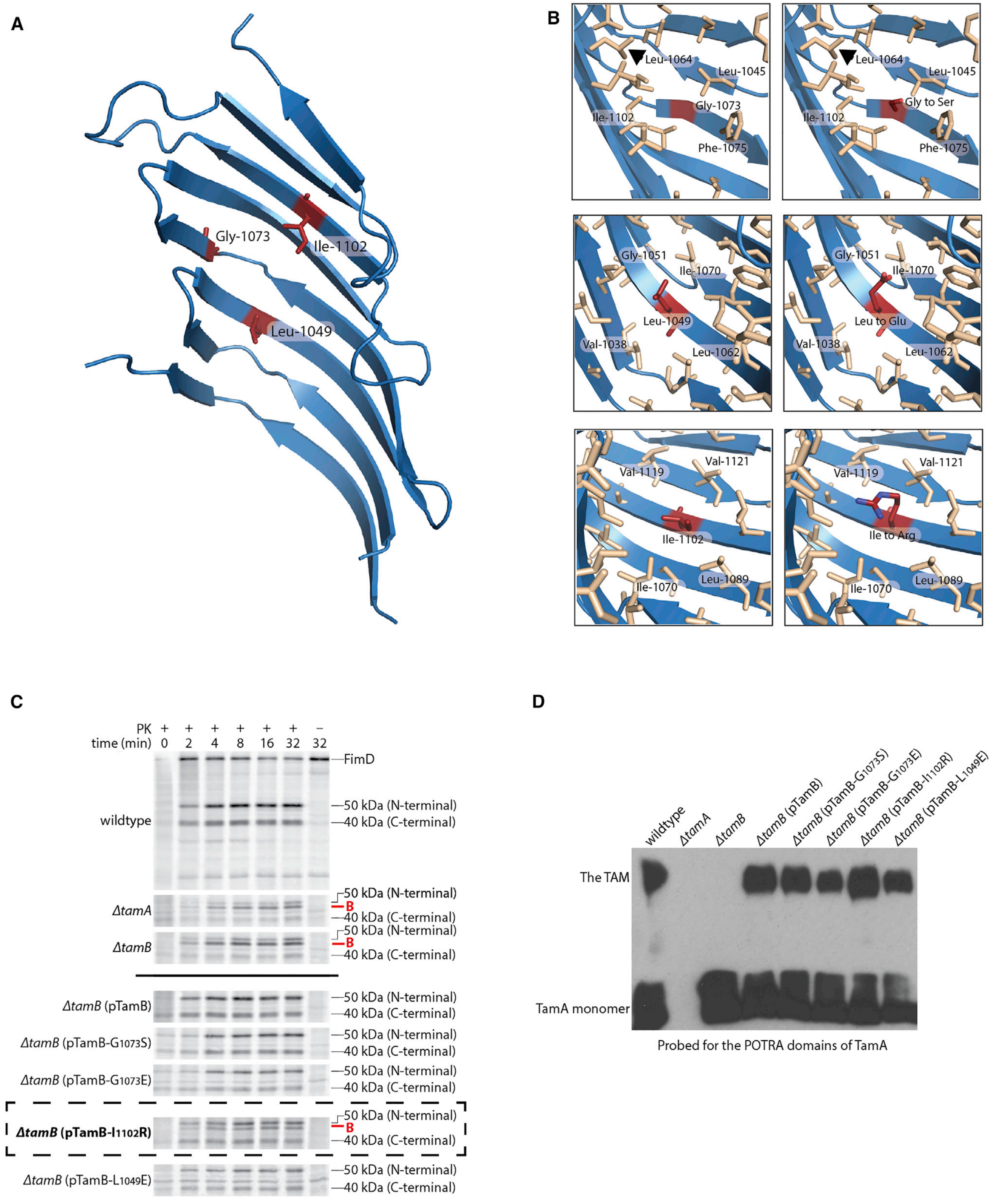

Figure 4. The Effect on the Function of TamB of the Introduction of Charged Residues into the Hydrophobic β Taco of $\text{TamB}_{963\text{-}1138}$

(A) Positions of substitution of hydrophobic residues in the $\text{TamB}_{963\text{-}1138}$ β taco (shown as red sticks). Glycine at position 1,073 is conserved with SSG4.

(B) The local environment of the hydrophobic amino acid changed (panel 1) and their corresponding charged residue substitutions (panel 2).

(C and D) The effect of the mutations shown in (A and B) on the ability of a plasmid-encoded copy of the *tamB* allele to complement a Δ*tamB* null-phenotype.

(legend continued on next page)

extracts revealed that both mutant versions of TamB were capable of interacting with TamA to form the TAM, indicating that the defect in TamB^{Ile1102Arg} is not due to a gross defect in TamB production or structure (Figure 4D). Why TamB^{Ile1102Arg} was defective in our assay, but TamB^{Leu1049Glu} remained functional, is unknown. However, while the Leu_{1049}Glu mutation would certainly change the local charge of the β taco, it does not project into the cavity to the extent that bulky arginine at 1,102 does. Future work involving more thorough mutagenesis studies of TamB would be useful in answering these questions.

To create the hydrophobic β taco structure found in $TamB_{963-1138}$, the amino acid sequence of the β strands consist of alternating hydrophobic and hydrophilic amino acids. The sidechains projecting from a face of a β sheet are on alternate sides of the strands, so that the patterning observed in β taco of TamB creates one hydrophobic face (the internal cavity) and one hydrophilic face that would face the periplasmic environment. This sequence pattern is reminiscent of β barrel membrane proteins but in that case the hydrophobic side of the β sheet is embedded in the lipid bilayer. Sequence analysis of the TamB family reveals this alternating pattern of conserved hydrophobic and hydrophilic residues occurs not only in the $TamB_{963-1138}$, but is widely distributed throughout the majority of TamB (Figure S4). Extrapolating from the structure of $TamB_{963-1138}$, this pattern suggests that the extended TamB molecule consists of long sections of hydrophobic channel. This proposed structure for TamB has a striking similarity to the well-characterized LPS transport system of Gram-negative bacteria (Sperandeo et al., 2009). Three proteins from this system, LptC, LptA, and LptD, contain or consist of a β jelly roll with an interior hydrophobic groove (Dong et al., 2014). These proteins are predicted to interact to form a hydrophobic conduit for the aliphatic chains of LPS across the periplasm, from the inner to OMs (Bollati et al., 2015; Dong et al., 2014). In an interesting parallel to $TamB_{963-1138}$, the β jelly domain of LptD, the OM component of this system, was crystallized with two detergent molecules in its hydrophobic groove (Qiao et al., 2014).

A Structure-Function Relationship in Distant DUF490 Homologs?

TamB homologs have been shown to be widely conserved in bacterial diderms, where they are involved in OM biogenesis in distantly related genera, from *Escherichia* to *Borrelia* to *Deinococcus* (Heinz et al., 2015; Iqbal et al., 2016; Selkrig et al., 2015; Yu et al., 2017). The distribution of TamB-like proteins is, however, not limited to the bacterial kingdom, with proteins containing the conserved DUF490 domain having also been identified in plants (Heinz et al., 2015). In a recent study screening rice (*Oryza sativa*) mutants for defects in starch accumulation, the protein SSG4 (for substandard starch grain 4) was identified. SSG4 is a large (2,132 amino acid) protein consisting of predominantly β structure and a TamB-like C-terminal DUF490 domain. SSG4 is localized to the amyloplast, the plastid responsible for starch synthesis in plants. This organelle was derived by evolution from an ancient symbiotic Cyanobacterium (Chan et al., 2011). Mutation of Gly_{1924}Ser in the DUF490 domain of SSG4 leads to enlarged starch granules and seed chalkiness (Matsushima et al., 2014). The authors suggest that this glycine is crucial to function and that it is conserved in TamB proteins from Proteobacteria (Matsushima et al., 2014). While plastids and Cyanobacteria share an evolutionary history, their protein-transport pathways are not homologous: proteins are imported into plastids from the cytoplasm, and there is no evidence of a vestigial protein secretion pathway from the internal compartments of the plastid out to its OM (Inoue, 2011; Strittmatter et al., 2010). Therefore, if SSG4 also plays a role in membrane protein biogenesis in the plastid it must be distinct from that of TamB.

Sequence alignment between TamB and SSG4 shows that the conserved glycine falls within the $TamB_{963-1138}$ crystal structure corresponding to Gly_{1073} (Matsushima et al., 2014). Gly_{1073} is located in β strand 4, adjacent to the kink in the β sheet caused by Pro_{1071} (Figure 4A). To test the significance of glycine at this position for the function of TamB, we subjected it to mutagenesis. However, substitution of either serine or glutamate for Gly_{1073} did not affect the function of the TAM in the assembly of FimD into the OM of *E. coli* (Figures 4C and 4D). While this finding does not rule out the importance of Gly_{1073} in the function of TamB, it shows that substitution of this residue does not result in a gross defect in the function of this protein. To determine if TamB and SSG4 do indeed share a related function in these distantly related organisms, further investigation will be required.

STAR★METHODS

Detailed methods are provided in the online version of this paper and include the following:

- KEY RESOURCES TABLE
- CONTACT FOR REAGENT AND RESOURCE SHARING
- EXPERIMENTAL MODEL AND SUBJECT DETAILS
- METHOD DETAILS
  - Protein Expression, Purification, Crystallization and Data Collection
  - Size-Exclusion Chromatography Multiangle Light Scattering (SEC-MALS)
  - Circular Dichroism Analysis
  - Experimental Phasing, Model Building and Refinement

(C) Pulse-chase assessment of ^{35}S-FimD assembly was monitored over time in wild-type, Δ*tamA*, or Δ*tamB* cells. Each strain carried pKS02 (for *fimD* expression) and either the control pACYCDuet-1 plasmid, or the indicated complementing *tamB* plasmid. Aliquots were taken at the indicated timepoints and treated with or without 50 μg/mL proteinase K (PK). Total protein was analyzed by SDS-PAGE and storage phosphor imaging. The presence of the 45 kDa fragment B (labeled in red), is indicative of improperly folded FimD due to impaired functioning of the TAM. The defect observed in the complementation of Δ*tamB* with pTamB-I_{1102}R is highlighted with a dashed rectangle.

(D) Membrane extract of wild-type, Δ*tamA*, or Δ*tamB* cells harboring either the control pACYCDuet-1 plasmid, or the indicated complementing *tamB* plasmid, were prepared. Membrane protein (100 μg) was analyzed by blue native (BN)-PAGE and immunoblotting, using an antibody raised to the N-terminal POTRA domains of TamA (Selkrig et al., 2012). The TAM does not form in Δ*tamA* or Δ*tamB* mutants. All alleles of *tamB* restore a wild-type phenotype to the TAM behavior on BN-PAGE.

○ Sequence and Structure Analysis
○ TamB Plasmid Mutagenesis
○ Chemical Transformation
○ Electro-Transformation
○ Crude Membrane Isolation
○ Blue Native-PAGE and Immunoblotting
○ Pulse Chase Analysis of FimD Assembly
● QUANTIFICATION AND STATISTICAL ANALYSIS
● DATA AND SOFTWARE AVAILABILITY

SUPPLEMENTAL INFORMATION

Supplemental Information includes four figures and one table and can be found with this article online at https://doi.org/10.1016/j.str.2017.10.002.

AUTHOR CONTRIBUTIONS

R.G., C.J.S., I.J., D.W., and K.M. conceived and designed the experiments; R.G., C.J.S., G.V., and I.J. performed the experiments; R.G., C.J.S., G.V., I.J., D.W., and K.M. analyzed the data; R.G., T.L., and D.W. contributed reagents/materials/analysis tools; R.G., C.J.S., T.L., I.J., and D.W. wrote the paper.

ACKNOWLEDGMENTS

We thank the Diamond Light Source for access to beamlines I02, I04-1, and I24 (proposal numbers MX6638 and MX8659). The work was funded by the NHMRC Program in Cellular Microbiology (1092262). R.G. was funded by a Sir Henry Wellcome Fellowship (award number 106077/Z/14/Z). During this work I.J. was supported by a studentship from the Wellcome Trust (award number 093592/Z/10/Z).

Received: May 30, 2017
Revised: August 31, 2017
Accepted: October 14, 2017
Published: November 9, 2017

REFERENCES

Adams, P.D., Afonine, P.V., Bunkoczi, G., Chen, V.B., Davis, I.W., Echols, N., Headd, J.J., Hung, L.-W., Kapral, G.J., Grosse-Kunstleve, R.W., et al. (2010). PHENIX: a comprehensive Python-based system for macromolecular structure solution. Acta Crystallogr. D Biol. Crystallogr. *66*, 213–221.

Afonine, P.V., Moriarty, N.W., Mustyakimov, M., Sobolev, O.V., Terwilliger, T.C., Turk, D., Urzhumtsev, A., and Adams, P.D. (2015). FEM: feature-enhanced map. Acta Crystallogr. D Biol. Crystallogr. *71*, 646–666.

Bakelar, J., Buchanan, S.K., and Noinaj, N. (2016). The structure of the β-barrel assembly machinery complex. Science *351*, 180–186.

Bollati, M., Villa, R., Gourlay, L.J., Benedet, M., Dehò, G., Polissi, A., Barbiroli, A., Martorana, A.M., Sperandeo, P., Bolognesi, M., et al. (2015). Crystal structure of LptH, the periplasmic component of the lipopolysaccharide transport machinery from *Pseudomonas aeruginosa*. FEBS J. *282*, 1980–1997.

Chan, C.X., Gross, J., Yoon, H.S., and Bhattacharya, D. (2011). Plastid origin and evolution: new models provide insights into old problems. Plant Physiol. *155*, 1552–1560.

Dong, H., Xiang, Q., Gu, Y., Wang, Z., Paterson, N.G., Stansfeld, P.J., He, C., Zhang, Y., Wang, W., and Dong, C. (2014). Structural basis for outer membrane lipopolysaccharide insertion. Nature *511*, 52–56.

Drozdetskiy, A., Cole, C., Procter, J., and Barton, G.J. (2015). JPred4: a protein secondary structure prediction server. Nucleic Acids Res. *43*, W389–W394.

Emsley, P., Lohkamp, B., Scott, W.G., and Cowtan, K. (2010). Features and development of Coot. Acta Crystallogr. D Biol. Crystallogr. *66*, 486–501.

Finn, R.D., Clements, J., and Eddy, S.R. (2011). HMMER web server: interactive sequence similarity searching. Nucleic Acids Res. *39*, W29–W37.

Gu, Y., Li, H., Dong, H., Zeng, Y., Zhang, Z., Paterson, N.G., Stansfeld, P.J., Wang, Z., Zhang, Y., and Wang, W. (2016). Structural basis of outer membrane protein insertion by the BAM complex. Nature *531*, 64–69.

Han, L., Zheng, J., Wang, Y., Yang, X., Liu, Y., Sun, C., Cao, B., Zhou, H., Ni, D., and Lou, J. (2016). Structure of the BAM complex and its implications for biogenesis of outer-membrane proteins. Nat. Struct. Mol. Biol. *23*, 192.

Heinz, E., Selkrig, J., Belousoff, M.J., and Lithgow, T. (2015). Evolution of the translocation and assembly module (TAM). Genome Biol. Evol. *7*, 1628–1643.

Heinz, E., Stubenrauch, C.J., Grinter, R., Croft, N.P., Purcell, A.W., Strugnell, R.A., Dougan, G., and Lithgow, T. (2016). Conserved features in the structure, mechanism, and biogenesis of the inverse autotransporter protein family. Genome Biol. Evol. *8*, 1690–1705.

Inoue, K. (2011). Emerging roles of the chloroplast outer envelope membrane. Trends Plant Sci. *16*, 550–557.

Iqbal, H., Kenedy, M.R., Lybecker, M., and Akins, D.R. (2016). The TamB ortholog of *Borrelia burgdorferi* interacts with the β-barrel assembly machine (BAM) complex protein BamA. Mol. Microbiol. *102*, 757–774.

Josts, I., Grinter, R., Kelly, S.M., Mosbahi, K., Roszak, A., Cogdell, R., Smith, B.O., Byron, O., and Walker, D. (2014). Recombinant expression, purification, crystallization and preliminary X-ray diffraction analysis of the C-terminal DUF490963–1138 domain of TamB from Escherichia coli. Acta Crystallogr. F Struct. Biol. Commun. *70*, 1272–1275.

Kabsch, W. (2010). XDS. Acta Crystallogr. D Biol. Crystallogr. *66*, 125–132.

Matsushima, R., Maekawa, M., Kusano, M., Kondo, H., Fujita, N., Kawagoe, Y., and Sakamoto, W. (2014). Amyloplast-localized substandard starch grain4 protein influences the size of starch grains in rice endosperm. Plant Physiol. *164*, 623–636.

McNicholas, S., Potterton, E., Wilson, K., and Noble, M. (2011). Presenting your structures: the CCP4mg molecular-graphics software. Acta Crystallogr. D Biol. Crystallogr. *67*, 386–394.

Noinaj, N., Kuszak, A.J., Gumbart, J.C., Lukacik, P., Chang, H., Easley, N.C., Lithgow, T., and Buchanan, S.K. (2013). Structural insight into the biogenesis of β-barrel membrane proteins. Nature *501*, 385–390.

Pizarro-Cerdá, J., and Cossart, P. (2006). Bacterial adhesion and entry into host cells. Cell *124*, 715–727.

Qiao, S., Luo, Q., Zhao, Y., Zhang, X.C., and Huang, Y. (2014). Structural basis for lipopolysaccharide insertion in the bacterial outer membrane. Nature *511*, 108–111.

Rollauer, S.E., Sooreshjani, M.A., Noinaj, N., and Buchanan, S.K. (2015). Outer membrane protein biogenesis in Gram-negative bacteria. Philos. Trans. R. Soc. Lond. B Biol. Sci. *370*, 20150023.

Selkrig, J., Mosbahi, K., Webb, C.T., Belousoff, M.J., Perry, A.J., Wells, T.J., Morris, F., Leyton, D.L., Totsika, M., Phan, M.-D., et al. (2012). Discovery of an archetypal protein transport system in bacterial outer membranes. Nat. Struct. Mol. Biol. *19*, 506–510.

Selkrig, J., Belousoff, M.J., Headey, S.J., Heinz, E., Shiota, T., Shen, H.-H., Beckham, S.A., Bamert, R.S., Phan, M.-D., and Schembri, M.A. (2015). Conserved features in TamA enable interaction with TamB to drive the activity of the translocation and assembly module. Sci. Rep. *5*, 12905.

Sheldrick, G. (2010). Experimental phasing with SHELXC/D/E: combining chain tracing with density modification. Acta Crystallogr. D Biol. Crystallogr. *66*, 479–485.

Shen, H.-H., Leyton, D.L., Shiota, T., Belousoff, M.J., Noinaj, N., Lu, J., Holt, S.A., Tan, K., Selkrig, J., and Webb, C.T. (2014). Reconstitution of a nanomachine driving the assembly of proteins into bacterial outer membranes. Nat. Commun. *5*, 5078.

Silhavy, T.J., Kahne, D., and Walker, S. (2010). The bacterial cell envelope. Cold Spring Harb. Perspect. Biol. *2*, a000414.

Sperandeo, P., Dehò, G., and Polissi, A. (2009). The lipopolysaccharide transport system of Gram-negative bacteria. Biochim. Biophys. Acta *1791*, 594–602.

Strittmatter, P., Soll, J., and Bölter, B. (2010). The chloroplast protein import machinery: a review. Methods Mol. Biol. *619*, 307–321.

Strong, M., Sawaya, M.R., Wang, S., Phillips, M., Cascio, D., and Eisenberg, D. (2006). Toward the structural genomics of complexes: crystal structure of a PE/PPE protein complex from *Mycobacterium tuberculosis*. Proc. Natl. Acad. Sci. USA *103*, 8060–8065.

Stubenrauch, C., Belousoff, M.J., Hay, I.D., Shen, H.-H., Lillington, J., Tuck, K.L., Peters, K.M., Phan, M.-D., Lo, A.W., and Schembri, M.A. (2016a). Effective assembly of fimbriae in *Escherichia coli* depends on the translocation assembly module nanomachine. Nat. Microbiol. *1*, 16064.

Stubenrauch, C., Grinter, R., and Lithgow, T. (2016b). The modular nature of the β-barrel assembly machinery, illustrated in *Borrelia burgdorferi*. Mol. Microbiol. *102*, 753–756.

Voulhoux, R., Bos, M.P., Geurtsen, J., Mols, M., and Tommassen, J. (2003). Role of a highly conserved bacterial protein in outer membrane protein assembly. Science *299*, 262–265.

Webb, C.T., Heinz, E., and Lithgow, T. (2012). Evolution of the β-barrel assembly machinery. Trends Microbiol. *20*, 612–620.

Winn, M.D., Ballard, C.C., Cowtan, K.D., Dodson, E.J., Emsley, P., Evans, P.R., Keegan, R.M., Krissinel, E.B., Leslie, A.G., and McCoy, A. (2011). Overview of the CCP4 suite and current developments. Acta Crystallogr. D Biol. Crystallogr. *67*, 235–242.

Wu, T., Malinverni, J., Ruiz, N., Kim, S., Silhavy, T.J., and Kahne, D. (2005). Identification of a multicomponent complex required for outer membrane biogenesis in *Escherichia coli*. Cell *121*, 235–245.

Yu, J., Li, T., Dai, S., Weng, Y., Li, J., Li, Q., Xu, H., Hua, Y., and Tian, B. (2017). A tamB homolog is involved in maintenance of cell envelope integrity and stress resistance of *Deinococcus radiodurans*. Sci. Rep. *7*, 45929.

STAR★METHODS

KEY RESOURCES TABLE

REAGENT or RESOURCE	SOURCE	IDENTIFIER
Antibodies		
Anti-TamA (POTRA domains only) antibody produced in rabbit	This paper	N.A
Anti-Rabbit IgG (whole molecule)-Peroxidase antibody produced in goat	Sigma-Aldrich	A6154; RRID: AB_258284
Bacterial and Virus Strains		
E. coli DH5α: F- Φ80lacZΔM15 Δ(lacZYA-argF) U169 recA1 endA1 hsdR17(rk-, mk+) phoA supE44 thi-1 gyrA96 relA1 λ-	Invitrogen	For example: Cat#, 18265017
E. coli BL21(DE3): F- ompT hsdSB (rB-mB-) gal dcm (DE3)	New England Biolabs	For example: Cat#, C2527H
E. coli BL21 Star™ (DE3): F- ompT hsdSB (rB-mB-) gal dcm rne131 (DE3)	Invitrogen	For example: Cat#, C6010-03
E. coli BL21 Star™ (DE3) ΔtamA: F- ompT hsdSB (rB-mB-) gal dcm rne131 (DE3) ΔtamA::Kan	Stubenrauch et al., 2016a	N.A
E. coli BL21 Star™ (DE3) ΔtamB::Kan	Stubenrauch et al., 2016a	N.A
Chemicals, Peptides, and Recombinant Proteins		
20 mg.mL-1 proteinase K solution	Promega	Cat#, MC500
EXPRE35S35S [35S]-Protein Labelling Mix	Perkin Elmer	NEG072
Amersham ECL Prime Western Blotting Detection Reagent	GE Healthcare Life Sciences	RPN2232
Dnase A	Sigma	Cat#, E1014
EDTA-free Complete Protease Inhibitor Cocktail	Roche	Cat#, 04693132001
Ni-NTA Agarose	Invitrogen	Cat#, R901-01
LDAO	Sigma	Cat#, 40236
Deposited Data		
Crystal Structure of TamB963-1138	This paper	PDB:5VTG
Oligonucleotides		
TamB Leu 1049 to Glu (TTTGGCCTGAAAGCGCGGGAGACGGGCGATCT CAATGT),	This paper	N.A
TamB Gly 1073 to Ser (GCAGATCAACATCCCTGAAAGTCGCTTCCATGC CTATGGTC)	This paper	N.A
TamB Gly 1073 to Glu (CAGATCAACATCCCTGAAGAGCGCTTCCATGCC TATGGTC)	This paper	N.A
TamB Ile 1102 to Arg (GCCAGATCAACCGTATCTTAATCGTGAAGCTATTC GTAACCCGGA)	This paper	N.A
Recombinant DNA		
pET21a, confers ampicillin resistance	Merck	69740-3
pACYCDuet-1, confers chloramphenicol resistance (used as an empty vector control for the various tamB complementation plasmids)	Novagen	Cat#, 71147-3
pTamB, confers chloramphenicol resistance	Stubenrauch et al., 2016a	referred to as pCJS72 therein
pTamB-G1073S, confers chloramphenicol resistance	This paper	N.A
pTamB-G1073E, confers chloramphenicol resistance	This paper	N.A
pTamB-I1102R, confers chloramphenicol resistance	This paper	N.A
pTamB-L1049E, confers chloramphenicol resistance	This paper	N.A
pKS02, confers ampicillin resistance	Stubenrauch et al., 2016a	N.A
Software and Algorithms		
Coot	Emsley et al., 2010	https://sbgrid.org/software/
CCP4 suite	Winn et al., 2011	https://sbgrid.org/software/
XDS	Kabsch, 2010	https://sbgrid.org/software/
Phenix	Adams et al., 2010	https://sbgrid.org/software/

(*Continued on next page*)

Continued		
REAGENT or RESOURCE	SOURCE	IDENTIFIER
Other		
Storage phosphor screen: Unmounted General Purpose, 20 × 25 cm	GE Healthcare Life Sciences	Cat# 63-0034-87
Exposure cassette for unmounted screens, 20 × 25 cm	GE Healthcare Life Sciences	Cat# 63-0035-44
Typhoon Trio	GE Healthcare Life Sciences	63-0055-87
SG50 gradient maker	GE Healthcare Life Sciences	SG50

CONTACT FOR REAGENT AND RESOURCE SHARING

Further information and requests for resources and reagents should be directed to and will be fulfilled by the Lead Contact, Rhys Grinter (Rhys.grinter@monash.edu).

EXPERIMENTAL MODEL AND SUBJECT DETAILS

Expression of proteins used for crystallographic studies and analytical size exclusion chromatography (SEC) was performed in *E. coli* BL21(DE3). Cells were grown at 37 °C in Terrific broth (TB). When optical density at 600 nm reached 0.8, protein expression was induced with the addition of 0.5 mM IPTG and cells were incubated overnight at 25 °C before harvest.

For membrane isolation, BN-PAGE and pulse chase analyses, *E. coli* BL21 Star™ (DE3) and derivative strains were used. For plasmid storage, *E. coli* DH5α was used. These strains were routinely grown in lysogeny broth (LB, containing 10 g.L^{-1} tryptone, 5 g.L^{-1} yeast extract and 5 g.L^{-1} NaCl), at 37 °C and 200 strokes per minute (25 mm orbit). For strain storage, saturated overnight culture was diluted 1:1 in 40 % v/v glycerol, snap frozen in liquid nitrogen and kept at -80 °C.

Where appropriate, the following antibiotics were used for selection: 34 μg.mL^{-1} chloramphenicol, 30 μg.mL^{-1} kanamycin, and/or 100 μg.mL^{-1} ampicillin. If solid media was required, 15 g.L^{-1} agar was added to the growth medium.

METHOD DETAILS

Protein Expression, Purification, Crystallization and Data Collection

Native TamB$_{963-1138}$ was expressed and purified as described by (Josts et al., 2014). Briefly, the gene fragment encoding the DUF490 domain residues 963-1138 from TamB from *E. coli* K12 was ligated into pET-21a via NdeI and XhoI restriction sites producing a C-terminally His$_6$ tagged product. This construct was transformed into *E. coli* BL21 (DE3) cells which were grown in LB (+ 100 ug.ml^{-1} Ampicillin and 3% glycerol) to an OD of 0.6, before induction with 0.5 mM IPTG. Cells were then grown for 15 hours at 25°C and harvested by centrifugation (5000 g). Cells were resuspended in 20 mM Tris–HCl, 10 mM imidazole, 0.5 M NaCl, 5%(v/v) glycerol, 0.05% LDAO pH 7.5 then lysed via sonication, supernatant was clarified by centrifugation (30,000 g). TamB$_{963-1138}$ was purified from this clarified supernatant by a 2-step purification of nickel affinity and size exclusion (Superdex S200) chromatography. Clarified cell lysate was applied to a 5ml Ni-agarose column and the column was washed with at least 10 column volumes of 20 mM Tris–HCl, 10 mM imidazole, 0.5 M NaCl, 5%(v/v) glycerol, 0.05% LDAO pH 7.5. Protein was then eluted from the column with a 0-100% gradient of of 20 mM Tris–HCl, 500 mM imidazole, 0.5 M NaCl, 5%(v/v) glycerol, 0.05% LDAO pH 7.5 over 10 column volumes. Fractions containing DUF490$_{963-1138}$ were then applied to a 26/200 Superdex S200 column equilibrate in 20 mM Tris-HCl, 200 mM NaCl, 0.05% LDAO. DUF490$_{963-1138}$ eluted as multimeric species on size exclusion, however a single peak most likely corresponding to a monomer or dimer was pooled and concentrated to 8-15 mg.ml^{-1} prior sparse matrix screening for crystallization conditions.

For selenomethionine labelling TamB$_{963-1138}$ expression construct described above was transformed into the methionine auxotrophic strain *E. coli* B834 (DE3). Cells were grown at 37 °C in M9 minimal media (+ 100 ug.ml^{-1} ampicillin, 50 ug.ml^{-1} selenomethionine, 100 ug.ml^{-1} other amino acids, 0.5 ug.ml thiamine) to an OD$_{600}$ of 0.4 before induction with 0.5 mM IPTG. Cells were then grown for 15 hours at 25 °C before harvesting, and protein purified as described above. 1 mM DTT was included in all buffers to prevent oxidation of the selenium.

Crystallisation was performed as previously described (Josts et al., 2014). Protein for crystalisation was in a buffer containing: 50 mM Tris–HCl, 200 mM NaCl, 0.05% LDAO pH 7.5. Crystals were grown with a reservoir solution containing: 0.1 M HEPES, 15%(v/v) PEG 400, 0.2 M CaCl2 pH 7.0. Crystals were transferred to cryoprotectant consisting of reservoir solution with 25%(v/v) PEG 400 and flash cooled in liquid nitrogen. Data was collected at 100 °K (0.9752 Å) at Diamond Lightsource, UK.

Size-Exclusion Chromatography Multiangle Light Scattering (SEC-MALS)

The absolute molecular mass of TamB$_{963-1138}$ was determined by SEC-MALS. 100-μl protein samples (1-5 mg.ml^{-1}) were loaded onto a Superdex 200 10/300 GL size-exclusion chromatography column in 20 mM Tris , 200 mM NaCl 0.05 % LDAO [pH 7.9] at 0.6 ml/min with a Shimadzu Nexera SR. The column output was fed into a DAWN HELEOS II MALS detector (Wyatt Technology) followed by an Optilab T-rEX differential refractometer (Wyatt Technology). Light scattering and differential refractive index data were collected and

analyzed with ASTRA 6 software (Wyatt Technology). Molecular masses and estimated errors were calculated across individual eluted peaks by extrapolation from Zimm plots with a dn/dc value of 0.1850 ml/g. SEC-MALS data are presented with light scattering (LS) and refractive index change plotted alongside fitted molecular masses (Mr).

Circular Dichroism Analysis

Circular dichorism measurements were obtained for $TamB_{963-1138\ at}$ 1 mg/ml in 20 mM Tris , 200 mM NaCl [pH 7.9] the presence and absence of 0.03 % LDAO at 24°C using a Jasco J-810 spectropolarimeter (Jasco UK Ltd).

Experimental Phasing, Model Building and Refinement

Based on the Matthews coefficient for the $DUF490_{963-1138}$ crystals, two molecules were predicted to be present in the crystal asymmetric unit (ASU), with a solvent content of 50 %. One molecule per ASU was also a possibility, with a solvent content of 76 %. Each $DUF490_{963-1138}$ molecule has 2 methionine residues (discounting the N-terminal methionine which is likely to be cleaved), giving 4 as the most likely number selenium atoms present. To locate heavy atom sites in diffraction data from the selenomethionine labelled $DUF490_{963-1138}$ data was collected at the selenium edge and processed to 2.7 Å. Anomalous signal for the data was detected up to 7.4 Å using Xtriage from the Phenix package (Adams et al., 2010). This was weaker than expected given the methionine to amino acid residue ratio (1:88). ShelxC was employed for data preparation, followed by ShelxD to locate selenium sites (Sheldrick, 2010). The best substructure solutions were obtained with 3 selenium sites with occupancies of 0.87, 0.47 and 0.31, rather than the 4 sites expected for 2 molecules per ASU. These sites were then provided along with the DUF490 anomalous dataset to Autosol from the Phenix package for phasing and density modification (Adams et al., 2010). Contrast of the initial experimentally phased maps was poor, making it difficult to determine the corrected hand of the screw axis ($P3_121$ or $P3_221$). However, density modification greatly improved map contrast with clear density present for molecules consisting of an elongated U-shaped β-sheet in the solution from the correct hand with the space group $P3_221$ (Figure S1). This experimentally phased map was then used to construct a provisional model. This structure was then used as a molecular replacement model for the higher resolution native data (2.1 Å). The $DUF490_{963-1138}$ was then iteratively built and refined using COOT and Phenix refine to give the final structure with R_{work} and R_{free} of 20.8% a 25.1% respectively (Emsley et al., 2010).

Sequence and Structure Analysis

Structural analysis and figure construction was performed using pymol and QtMG structural graphics packages (McNicholas et al., 2011). Secondary structure prediction for TamB was performed using the JPred4 webserver (Drozdetskiy et al., 2015).

Amino acid sequences for TamB homologues were identified using a Hmmer search against the rp15 database, with TamB from *E. coli* as the query sequence and an e-value cut off of 1e-30. Sequences identified were triaged for those +/- 500 amino acids in length of TamB *from E. coli* and aligned using clustalx (Finn et al., 2011).

TamB Plasmid Mutagenesis

In order to introduce single amino acid mutations onto the $TamB_{963-1138}$ region of *tamB* in pTamB (pCJS72) the whole plasmid PCR mutagenesis method was utilised. A reaction was assembled in 50 μl H_2O containing: 2.5 U (1 μl) PfuTurbo polymerase, 5 μl 10 x Pfu reaction buffer, 125 ng each of forward and reverse primers (see below), 50 ng pTamB DNA and 1 μl 10 mM dNTP mix. The following forward primers were utilised for each mutation, with the reverse complement of the listed sequence used for the reverse primer:

Leu 1049 to Glu (TTTGGCCTGAAAGCGCGGGAGACGGGCGATCTCAATGT),
Gly 1073 to Ser (GCAGATCAACATCCCTGAAAGTCGCTTCCATGCCTATGGTC),
Gly 1073 to Glu (CAGATCAACATCCCTGAAGAGCGCTTCCATGCCTATGGTC),
Ile 1102 to Arg (GCCAGATCAACCGTATCTTAATCGTGAAGCTATTCGTAACCCGGA)

The reaction mixture was subjected to the following thermocycling regime: 1 x 95°C for 30 seconds, 18 x (95°C for 30 seconds, 55°C for 60 seconds, 68°C for 7 minutes). 1 μl of DpnI was then added to the reaction which was incubated at 37°C for 1 hour. The reaction mixture was then transformed into *E. coli* DH5α and plated onto LB agar containing 30 μg/ml chloramphenicol. Plasmid DNA was extracted from resultant colonies and sequenced to confirm that the desired mutation and no other mutations were present.

Chemical Transformation

E. coli DH5α were Saturated overnight cultures were diluted 1:50 into fresh 30 mL LB, supplemented with appropriate antibiotics, and incubated until mid-log phase. The culture was chilled on ice for 30 min, then subjected to centrifugation (4415 ×g, 4 °C, 15 min) and resuspended in 4.5 mL ice cold 0.1 M $CaCl_2$. The suspension was chilled on ice for a further 30 min, centrifuged as before and resuspended in 150 μL 0.1 M $CaCl_2$. Following a 2-hour incubation on ice, 75 μL LB (supplemented with 30 % w/v glycerol) were aliquoted and snap frozen and stored at -80 °C.

Cells (20-50 μL) were thawed on ice and incubated with 20-50 ng plasmid DNA for 40 min on ice. Cells were heat shocked at 42 °C for 45 s, then incubated on ice for 2 min before 250 μL LB media was added and cells were allowed to recover for 1 hour. Samples were then spread-plated onto LB agar containing appropriate antibiotics, and following a 24-hour incubation at 37 °C, transformants were selected for subsequent analyses.

Electro-Transformation

Saturated overnight cultures were diluted 1:50 into fresh 30 mL LB, supplemented with appropriate antibiotics, and incubated until mid-log phase. The culture was subjected to four rounds of centrifugation (3485 ×g, 4 °C, 10 min), followed by resuspension in increasingly smaller volumes of 10% v/v glycerol: 12 mL, 6 mL, 3 mL, 0.3 mL. Cells (50 μL) were briefly incubated on ice with 20-50 ng plasmid DNA and then transferred to a chilled electroporation cuvette (1 mm gap). Samples were electroporated (1.8 kV, 200 Ω, 25 μF) and immediately transferred to 250 μL LB and allowed to recover for 1 hour. Transformants were then selected for on solid media, supplemented with appropriate antibiotics, after a 24-hour incubation at 37 °C.

Crude Membrane Isolation

Saturated overnight cultures were diluted 1:100 into fresh 50 mL LB, supplemented with appropriate antibiotics, and incubated until the optical density at 600 nm was between 0.8 and 1.2. The culture was subjected to centrifugation (4609 ×g, 4 °C, 10 min) and then resuspended in 10 mL sonication buffer (2 mM EDTA, 150 mM NaCl, 10 mM Tris-HCl, pH 7.5). Samples were lysed by sonication and the sample was subjected to centrifugation to remove unbroken cells (2668 ×g, 4 °C, 5 min). The supernatant was then subjected to centrifugation (16743 ×g, 4 °C, 10 min), and the membrane pellet was resuspended in 1 mL SEM buffer (1 mM EDTA, 250 mM sucrose, 10 mM MOPS-KOH, pH 7.2). Membranes were snap frozen in liquid nitrogen, and stored at -80 °C.

Blue Native-PAGE and Immunoblotting

Membranes comprising 150 $\mu g.\mu L^{-1}$ protein were thawed on ice, and subjected to centrifugation (13600 ×g, 4 °C, 5 min). Membranes were resuspended in 36 μL blue native lysis buffer (10 $mg.mL^{-1}$ DDM, 1 mM PMSF, 50 mM NaCl, 50 mM 6-aminohexanoic acid, 1 mM EDTA, 7.5 % w/v glycerol, 25 mM imidazole-HCl, pH 7.0) (Note: PMSF has a short half-life in aqueous solutions and was therefore added immediately before use from a master stock of 100 mM PMSF in isopropanol). Samples were incubated on ice for no more than 30 min, and then subjected to centrifugation (21200 ×g, 4 °C, 10 min). The supernatant was transferred to 9 μL 5× blue native sample buffer (3 parts blue native lysis buffer and 1 part of a stock solution comprising 100 mM 6-aminohexanoic acid and 4 % w/v coomassie brilliant blue G-250). With a 40 % T, 2.6 % C acrylamide/bis acrylamide solution (such as 40% Acrylamide/bis solution 37.5:1, Bio-Rad, cat#1610148), a 4 % acrylamide (4 % v/v acrylamide 37.5:1 mixture, 3.75 % w/v glycerol, 500 mM 6-aminohexanoic acid, 25 mM imidazole-HCl, pH 7.0, 0.1 % v/v TEMED, 0.09 % w/v ammonium persulfate) and a 14 % acrylamide (14 % v/v acrylamide 37.5:1 mixture, 20.25 % w/v glycerol, 500 mM 6-aminohexanoic acid, 25 mM imidazole-HCl, pH 7.0, 0.05 % v/v TEMED, 0.045% w/v ammonium persulfate) mixture were used to cast a 4-14 % blue native gradient gel with an SG50 gradient maker as per manufacturer's instructions.

Samples (30 μL, comprising 100 μg membrane protein) and size markers (30 μL, 1.5 $\mu g.\mu L^{-1}$ ferritin, 1.5 $\mu g.\mu L^{-1}$ catalase, 1.5 $\mu g.\mu L^{-1}$ BSA, 50 mM NaCl, 55 mM 6-aminohexanoic acid, 1 mM EDTA, 7.5 % w/v glycerol, 0.2 % w/v Coomassie brilliant blue G-250, 25 mM imidazole-HCl, pH 7.0) were loaded onto 4-14 % blue native gradient gels and analysed by blue native PAGE as follows. Anode buffer (25 mM imidazole-HCl, pH 7.0) and dark blue cathode buffer (0.05 % w/v Coomassie brilliant blue G-250, 50 mM tricine-imidazole, pH 7.0) were added to the lower and upper tanks, respectively, and subjected to electrophoresis (250 V, 7.5-8.0 mA, 4 °C), until the dye front has migrated two-thirds of the gel. Replace the buffer in the upper tank with a slightly blue cathode buffer (0.001 % w/v Coomassie brilliant blue G-250, 50 mM tricine-imidazole, pH 7.0) and subject to electrophoresis (250 V, 7.5-8.0 mA, 4 °C) until the dark blue cathode buffer within the gel has been completely replaced by the slightly blue cathode buffer. Samples in the gel were denatured as follows. Blue native denaturing buffer (1 % w/v DTT, 4 % w/v SDS, 50 mM Tris-HCl, pH 6.8) was heated to 65 °C and poured over the 4-14 % blue native gradient gel (100 mL per 15 × 7 cm gel). The gel was then incubated (37 °C, 40 rpm, 25 mm orbit) for 20 min, and after briefly rinsing the gel in water, it was transferred to CAPS western transfer buffer (10 % v/v methanol, 20 mM CAPS-NaOH, pH 11.0) and incubated (room temperature, 40 rpm, 25 mm orbit) for 10 min. Denatured protein was transferred to 0.45 μm PVDF membranes (as per manufacturer's instructions) using CAPS western transfer buffer.

Residual coomassie was removed from the PVDF membrane (with methanol), before rinsing briefly in TBS-T buffer (0.2 % v/v Tween-20, 8 $g.L^{-1}$ NaCl, 0.2 $g.L^{-1}$ KCl, 30 $g.L^{-1}$ Tris-HCl, pH 7.4). Membranes were incubated in blocking buffer (2.5 % w/v skim milk powder in TBS-T) for 30-60 min (room temperature, 40 rpm, 25 mm orbit) or overnight (4 °C, 40 rpm, 25 mm orbit), before incubation in rabbit anti-TamA antibodies (1:20,000 dilution in blocking buffer) for 1 hour (room temperature, 40 rpm, 25 mm orbit). Membranes were washed three times in TBS-T (room temperature, 40 rpm, 25 mm orbit) for 5-10 min each, before incubation in goat anti-rabbit antibodies (1:20,000 dilution in blocking buffer) for 30 min (room temperature, 40 rpm, 25 mm orbit). Membranes were then washed as before, followed by incubation with Amersham ECL Prime Western Blotting Detection Reagent as per manufacturer's instructions. Chemiluminescent membranes were then exposed to super RX-N film (Fujifilm) in an Amersham Hypercassette™ (GE Healthcare Life Sciences) for up to 10 min, and developed using the SRX-101A medical film processor (Konica) as per manufacturer's instructions.

Pulse Chase Analysis of FimD Assembly

Saturated overnight cultures were diluted 1:100 into fresh LB, supplemented with chloramphenicol and ampicillin, and incubated until mid-log phase. The culture was subjected to centrifugation (4609 ×g, 4 °C, 10 min), washed in M9-S media (47.8 mM Na_2HPO_4, 22 mM KH_2PO_4, 8.56 mM NaCl, 11.1 mM glucose, 1.12 mM thiamine, 1 mM $MgCl_2$, 0.1 mM $CaCl_2$ and 45.4 $pg.mL^{-1}$ of the 18 standard non-sulphur containing amino acids, with an additional 181.6 $pg.mL^{-1}$ leucine [i.e. 227 $pg.mL^{-1}$ leucine total]), and after another

round of centrifugation, was resuspended in M9-S media (equal to the volume of LB removed following centrifugation). After 30 min incubation (37 °C, 200 rpm, 25 mm orbit), cells were normalised to an optical density at 600 nm of 0.6 and diluted 1:1 in 40 % v/v glycerol. The samples were then snap-frozen (1.3 mL aliquots) in liquid nitrogen and stored at -80 °C. Each batch of cells was considered to be one set of technical replicates.

Aliquots were thawed on ice, subjected to centrifugation (3000 ×g, 4 °C, 5 min) and resuspended in 650 μL M9-S media. Rifampicin (500 μg mL^{-1}) was added to inhibit transcription (37 °C, 400 rpm, 3 mm orbit) for 60 min before 0.2 mM of pre-warmed IPTG was added (30 °C, static) to induce pKS02-based *fimD* expression for 5 min. Cells were then 'pulsed' with 22 μCi.mL^{-1} of pre-warmed EXPRE^{35}S^{35}S [^{35}S]-Protein Labelling Mix (routinely containing 73 % [^{35}S]-methionine, 22 % [^{35}S]-cysteine) for 45 s, then immediately transferred to ice. Samples were then subjected to centrifugation (3000 ×g, 4 °C, 5 min) and resuspended in 650 μL M9+S media (M9-S media, but 1 mM $MgSO_4$ replaces $MgCl_2$ and 45.5 pg.mL^{-1} methionine and cysteine were added). The 'chase' component was considered to have begun immediately on resuspension of M9+S media and was performed for 32 minutes (30 °C, static).

For analysis by protease shaving, at each chase time point (10 sec, 2, 4, 8, 16, and 32 min), 50 μL aliquots were incubated on ice for 10 min with (all time points) or without (32 min timepoint only) 50 μg/mL proteinase K. Trichloroacetic acid (10 % v/v final) was then added and protein precipitates were collected by centrifugation (25000 ×g, 4 °C, 15 min). The precipitate was washed with acetone, subjected to centrifugation as before, and the pellet was air-dried. The sample was resuspended in 50 μL SDS sample buffer (10 % v/v glycerol, 1 % w/v SDS, 100 mM DTT, 0.01 % w/v bromophenol blue, 100 mM Tris-HCl, pH 6.8) and boiled for 3-5 min. Samples (10 μL) were loaded into 12% SDS acrylamide gel and analysed by SDS-PAGE. Proteins were transferred to 0.45 μm nitrocellulose membrane and the membrane was air dried. Radiation was captured for 12-18 hours using the storage phosphor screen (as per manufacturer's instructions) and analysed using the Typhoon Trio (320 nm).

QUANTIFICATION AND STATISTICAL ANALYSIS

Statistical methods were not utilised in analysis of the significance of data in this study

DATA AND SOFTWARE AVAILABILITY

The coordinates and structure factors for the crystal structure of $TamB_{963-1138}$ have been deposited in the PDB under the accession number: 5VTG

Structure, Volume *25*

Supplemental Information

The Structure of a Conserved Domain of TamB Reveals a Hydrophobic β Taco Fold

Inokentijs Josts, Christopher James Stubenrauch, Grishma Vadlamani, Khedidja Mosbahi, Daniel Walker, Trevor Lithgow, and Rhys Grinter

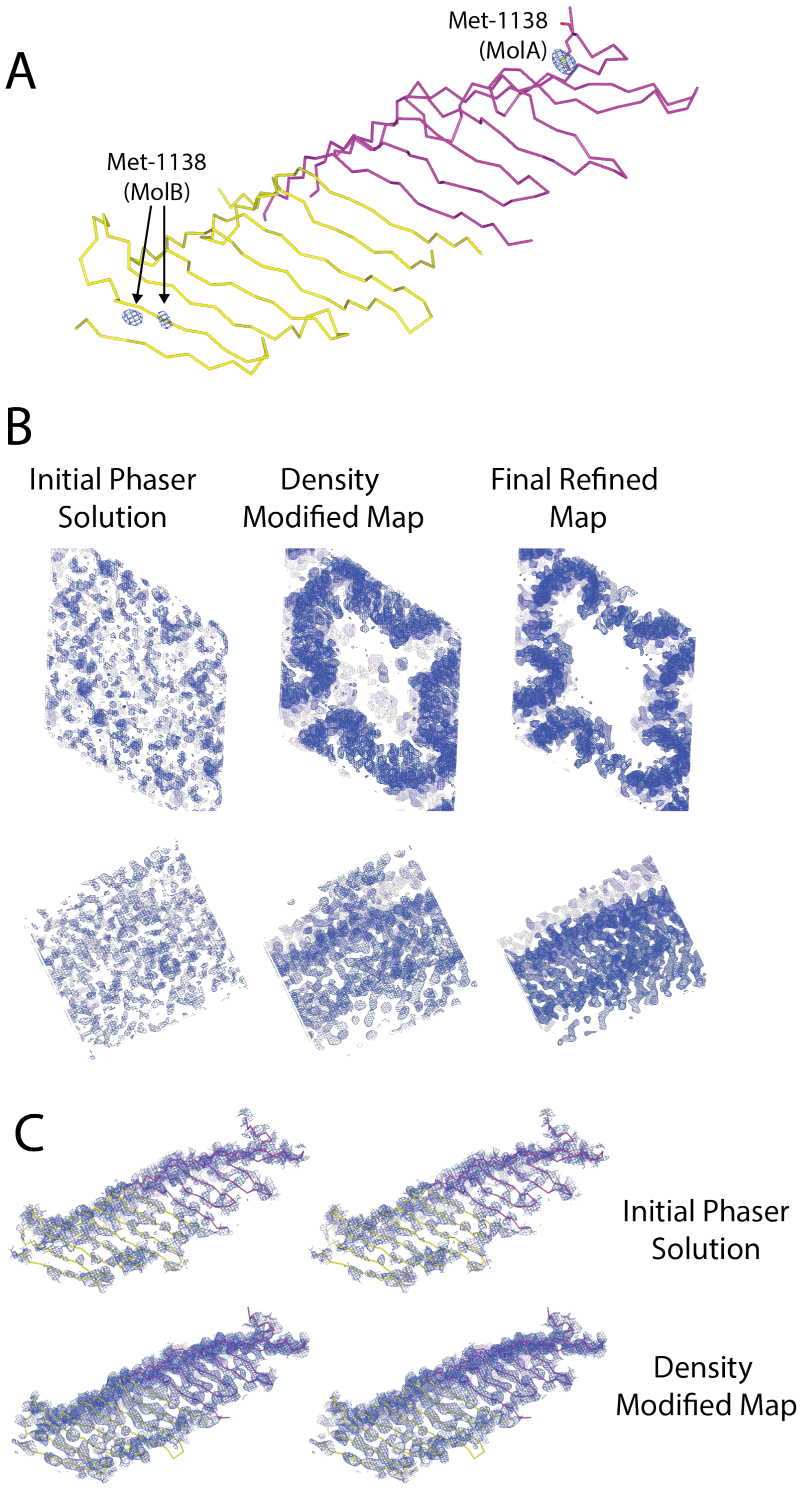

Figure S1, related to Figure 2: Electron density maps from TamB$_{963-1138}$ experimental phasing. (A) positions of selenium sites using for SAD phasing, the two sites attributable to methionine 1138 from molecule B are a result of partial occupancy of this residue. (B) and (C) TamB$_{963-1138}$ electron density maps pre and post density modification, model building and refinement.

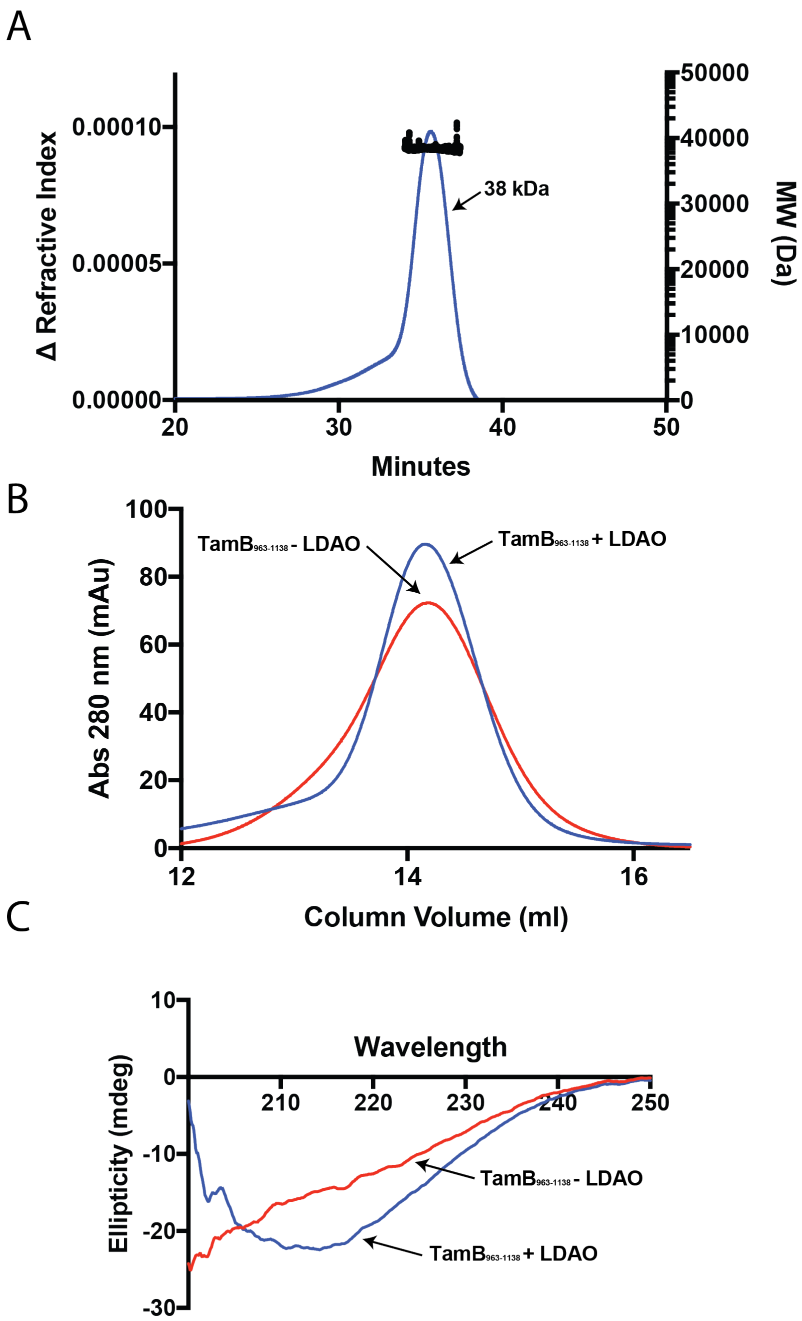

Figure S2, related Figures 2 and 3: Characterisation of TamB$_{963\text{-}1138}$ in solution. (A) SEC-MALS analysis of purified TamB$_{963\text{-}1138}$ showing that the protein exists as 38 kDa species corresponding to a dimer. (B) Analytical SEC of TamB$_{963\text{-}1138}$ in the presence and absence of 0.03 % LDAO. Both proteins elute at a volume corresponding to a dimer. (C) Circular Dichroism analysis of TamB$_{963\text{-}1138}$ In the presence and absence of LDAO. In the presence of LDAO TamB$_{963\text{-}1138}$ has a minimal ~218 nm, indicative of predominantly β-structure. In the absence of LDAO TamB$_{963\text{-}1138}$ has no minima in the far UV range (200-250 nm) suggesting partial disorder.

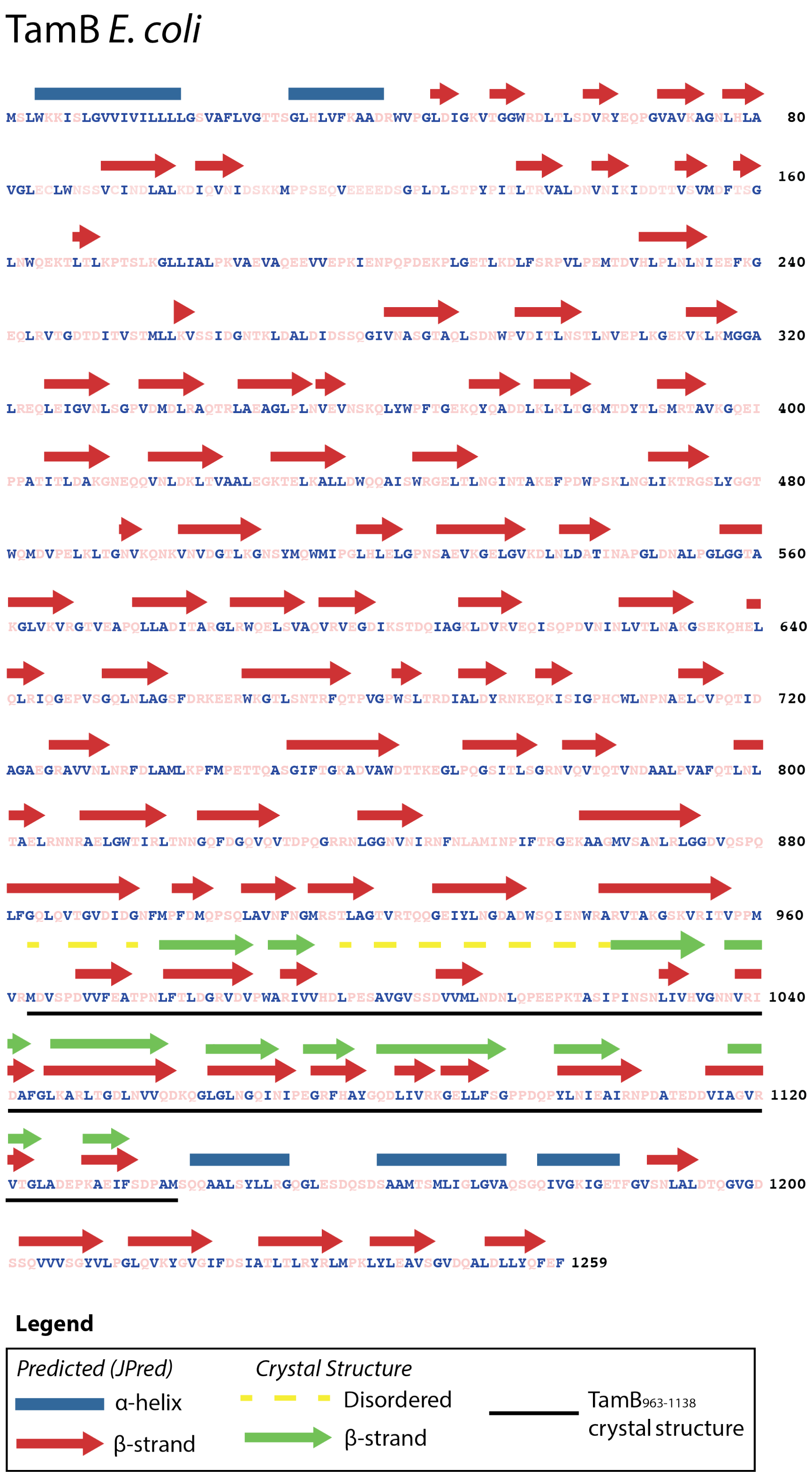

Figure S3, related to Figure 1: Predicted secondary structure of TamB from *E. coli*. TamB secondary structure; Predicted using JPred β-sheet = red arrow, α-helix = blue rectangle. From $TamB_{963-1138}$ crystal structure β-sheet = green arrow, disordered peptide = yellow broken line. The extent of the crystal structure is shown with black underline

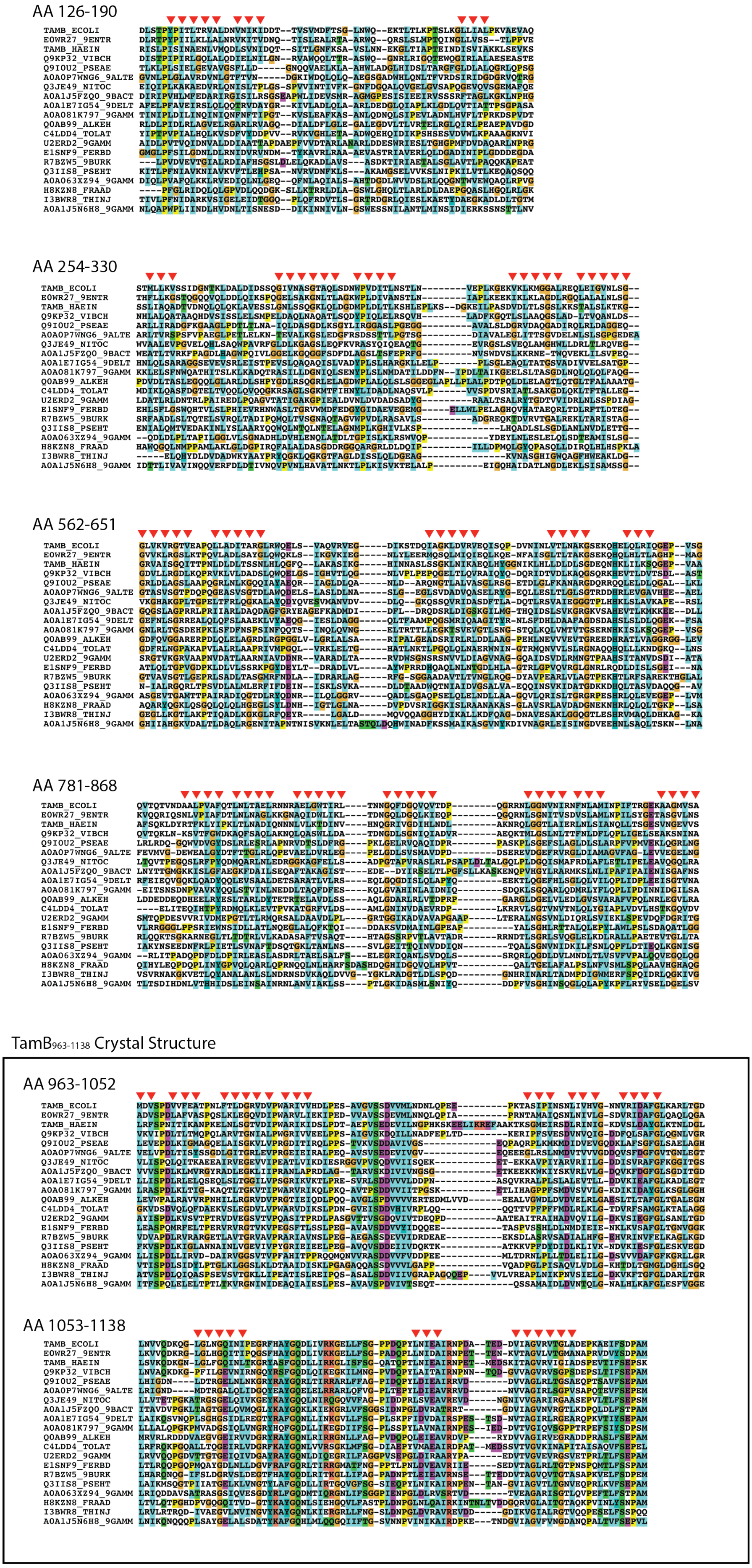
AA 126-190
AA 254-330
AA 562-651
AA 781-868
TamB963-1138 Crystal Structure
AA 963-1052
AA 1053-1138

Figure S4, related to Figure 3: Sequence alignment of sections of TamB homologues showing conserved pattern of alternating hydrophobic residues. See table S1 for details of sequences used for the alignment.

**Identifier**	**Organism**	**Length (AA)**	**E-Value**
TAMB_ECOLI	*Escherichia coli (strain K12)*	1259	1.10E-303
E0WR27_9ENTR	*Candidatus Regiella insecticola LSR1*	1253	3.70E-186
Q9KP32_VIBCH	Vibrio cholerae serotype O1 (strain ATCC 39315 / El Tor Inaba N16961)	1254	3.30E-132
R7BZW5_9BURK	*Sutterella sp. CAG:397*	1273	7.00E-119
TAMB_HAEIN	*Haemophilus influenzae (strain ATCC 51907 / DSM 11121 / KW20 / Rd)*	1298	6.70E-118
A0A081K797_9GAMM	*Endozoicomonas elysicola*	1298	2.50E-97
A0A1E7IG54_9DELT	*Desulfuromonadales bacterium C00003093*	1241	1.40E-94
Q3IIS8_PSEHT	*Pseudoalteromonas haloplanktis (strain TAC 125)*	1225	8.20E-92
Q0AB99_ALKEH	*Alkalilimnicola ehrlichii (strain ATCC BAA-1101 / DSM 17681 / MLHE-1)*	1283	7.20E-83
C4LDD4_TOLAT	*Tolumonas auensis (strain DSM 9187 / TA4)*	1240	1.40E-74
A0A1J5N6H8_9GAMM	*Gammaproteobacteria bacterium MedPE*	1278	3.60E-73
Q9I0U2_PSEAE	Pseudomonas aeruginosa (strain ATCC 15692	1221	8.70E-73
E1SNF9_FERBD	*Ferrimonas balearica (strain DSM 9799 / CCM 4581 / PAT)*	1264	1.60E-72
A0A0P7WNG6_9ALTE	*Marinobacter sp. HL-58*	1246	2.10E-64
Q3JE49_NITOC	*Nitrosococcus oceani (strain ATCC 19707)*	1262	1.20E-52
I3BWR8_THINJ	*Thiothrix nivea (strain ATCC 35100)*	1198	6.10E-52
U2ERD2_9GAMM	*Salinisphaera shabanensis E1L3A*	1297	2.10E-50
A0A1J5FZQ0_9BACT	*Nitrospirae bacterium CG2_30_53_67*	1249	2.30E-46
A0A063XZ94_9GAMM	*Nitrincola lacisaponensis*	1202	6.80E-45
H8KZN8_FRAAD	*Frateuria aurantia (strain ATCC 33424)*	1269	6.70E-42

Table S1, related to Figure 3: Details of TamB homologues used for sequence alignment. E-Value = similarity to sequence of TamB from *E. coli*.
